# Cardioprotective effect of resveratrol in myocardial ischemia/reperfusion injury and myocardial infarction: a pre-clinical meta-analysis in animal studies

**DOI:** 10.7717/peerj.20708

**Published:** 2026-02-04

**Authors:** Shi-Jie Wei, Qi-Hao Guo, Xin-Yu Wei, Si-Yu Chen, Sheng Deng, Liang An, Wen-Jing Zeng, Yi-Fan Zeng

**Affiliations:** 1 Department of Cardiovascular Surgery, the Second Xiangya Hospital, Central South University, Changsha, Hunan, China; 2 Department of Pharmacy, Xiangya Hospital, Central South University, Changsha, Hunan, China; 3 Department of Pharmacy, Shengjing Hospital of China Medical University, China Medical University, Shenyang, China; 4 National Clinical Research Center for Geriatric Disorders, Xiangya Hospital, Central South University, Changsha, Hunan, China; 5 The First Affiliated Hospital, Department of Pharmacy, Hengyang Medical School, University of South China, Hengyang, Hunan, China; 6 The Fourth Hospital of Baotou, Baotou, Neimenggu, China

**Keywords:** Resveratrol, Myocardial ischemia/reperfusion, Myocardial infarction, Meta-analysis, Pre-clinical evidence

## Abstract

This meta-analysis aimed to assess the cardioprotective effect of resveratrol (RES) against myocardial ischemia/reperfusion injury (MIRI) and myocardial infarction (MI) in the animal. PubMed, Web of Science, and EMBASE were searched. Primary outcomes included myocardial infarct size (IS) and cardiac function. Secondary outcomes included cardiac injury enzyme, oxidative stress level, inflammatory cytokine, and apoptosis rate. Subgroup analysis, publication bias, sensitivity analysis, meta-regression, and dosage-efficacy analysis were used to evaluate the risk of bias. Fifty-seven studies were included involving 1,125 animals. The results showed that RES treatment decreased IS in animal models of MIRI (SMD: −5.44; 95% CI [−6.42 to −4.45]; *P* < 0.01; I^2^ = 86%) and MI (SMD: −3.41; 95% CI [−4.44 to −2.38]; *P* < 0.01; I^2^ = 75%). Moreover, RES treatment improved cardiac function, decreased cardiac injury enzymes, down-regulated oxidative stress levels, alleviated inflammatory cytokine levels, and reduced apoptosis rate in animal models of MIRI and MI. This meta-analysis of preclinical animal studies suggested that RES may have potential in alleviating MIRI and MI. However, the translational potential of RES remains uncertain, and additional preclinical studies with standardized protocols, comorbid models, and eventual clinical trials are needed to confirm these results.

## Introduction

Cardiovascular diseases (CVDs) have become the leading cause of mortality throughout the world, with acute myocardial infarction (AMI) being the deadliest (Roth et al., 2020). AMI results from sudden coronary occlusion that causes severe and prolonged myocardial ischemia. The standard treatment strategy for rescuing ischemic myocardium is timely reperfusion therapy (such as PCI or thrombolysis) (Rao et al., 2025). Myocardial reperfusion therapy can timely and effectively restore the blood supply function of ischemic myocardial tissue, save ischemic myocardial cells, reduce myocardial infarction area, preserve left ventricular systolic function, and prevent heart failure (Jensen, Hjortbak & Bøtker, 2020; Wang et al., 2022). However, the restoration of blood flow itself may induce additional cellular injury, known as myocardial ischemia/reperfusion injury (MIRI), which aggravates irreversible apoptosis and necrosis of cardiomyocytes through Ca^2+^ overload, oxidative stress, mitochondrial dysfunction, inflammation, and cell acidosis (Ou et al., 2021; Jin et al., 2022; Shi et al., 2022; Xie et al., 2022; Chang et al., 2019). During the chronic phase, the necrotic myocardium is gradually replaced by fibrotic scar tissue, leading to chronic myocardial infarction (MI) characterized by irreversible loss of viable myocardium, ventricular remodeling, and progressive cardiac dysfunction (Frantz et al., 2022). Thus, AMI, MIRI, and chronic MI represent a continuous pathological spectrum from acute ischemia and reperfusion-induced injury to chronic structural remodeling and heart failure progression (Frantz et al., 2022; Schäfer et al., 2022). Experimental and clinical evidence highlights that the pathophysiology of acute MIRI and chronic MI differs substantially, and these differences are critical for interpreting preclinical studies and designing therapeutic interventions (Buja, 2023). Despite significant advances in reperfusion therapy and conventional pharmacological management, there is still no effective treatment specifically targeting MIRI or MI. Therefore, developing novel therapeutic strategies that can protect the myocardium from acute MIRI and chronic MI.

Natural nutraceuticals are primarily derived from foods and are considered less toxic than synthetic derivatives. Numerous studies have shown that natural nutraceuticals such as resveratrol (RES), curcumin, and anthocyanins have physiological benefits and protective effects against chronic diseases, which has also driven a surge in the use of nutritional foods and dietary supplements worldwide in recent years (Zeng et al., 2023; Cai et al., 2020; Li et al., 2024). RES, 3-4′-5-trihydroxystilbene, is a plant polyphenol found in multiple foods, including grapes, berries, plums, and peanuts (Acipreste Hudson et al., 2022; Raj et al., 2014). It is easy to be absorbed orally and excreted in urine and feces after metabolism. Studies have found that RES has multiple pharmacological effects, including anti-inflammatory, antioxidant, anticancer, cardiovascular protection, and neuroprotective effects (Gál et al., 2023). The prevention and treatment of MIRI and MI by RES are related to its anti-oxidative stress effect (Goh et al., 2007; Ananthakrishnan et al., 2009; Xing et al., 2021; Mao et al., 2019; Teimouri et al., 2022; Xuan et al., 2012). RES protects myocardial cells from MIRI not only by inhibiting superoxide levels and activating potassium channels (Goh et al., 2007), but also by significantly reducing the generation of reactive oxygen species (ROS) and inhibiting the opening of mitochondrial permeability transition (MPT) pores in the mouse heart (Ananthakrishnan et al., 2009). In addition, recent studies have found that RES may have a protective effect on MIRI through anti-oxidative stress and anti-inflammation mechanisms by analyzing various signaling pathways and serum biochemical substances of experimental animals (Rodrigo et al., 2022).

Therefore, to assess the role of RES against MIRI and MI, reconcile inconsistencies in preclinical findings, and advance its clinical translation potential, this study aimed to evaluate the cardioprotective effect of RES against MIRI and MI in animal studies.

## Materials & Methods

This meta-analysis has been registered in PROSPERO with identifier CRD42022383786.

### Search strategy

We searched PubMed, EMBASE, and Web of Science for relevant literature from inception to October 2025. The search strategy in PubMed was (“myocardial ischemia” OR “myocardial I/R” OR “myocardial I/R injury” OR “myocardial ischemia-reperfusion injury” OR “myocardial ischemia-reperfusion” OR “myocardial infarction”) AND (“resveratrol”). Additionally, the keyword above was used to modify the search strategy in EMBASE and Web of Science. This section was conducted by Yi-Fan Zeng and Qi-Hao Guo. The difference was resolved by Wen-Jing Zeng.

### Inclusion criteria

Inclusion criteria included: (1) Experimental models: blocking the left anterior descending (LAD) coronary artery, cardioplegia, or intravenously injecting vasoconstrictor, such as isoprenaline (ISO); (2) Treatment: RES was the only intervention compared with a control group, and (3) Data: detailed record the data on myocardial infarction size (IS) and/or other outcomes.

### Exclusion criteria

Exclusion criteria included: (1) abstracts or meeting posters, (2) review, (3) meta-analysis articles, (4) case report, (5) repeated literature, (6) no detailed data, (7) no animal model, and (8) without a control group.

### Data extraction

Two authors (Shi-Jie Wei, Xin-Yu Wei) independently screened eligible articles according to inclusion and exclusion criteria and extracted data using a standardized data extraction form. The authors then compared the results and resolved any differences after group discussion. The referee was Wen-Jing Zeng. Information extracted includes the authors, publication year, country, characteristics of the animal, sex of the animal, sample size, methods of anesthetic, model method, drug delivery method, and duration of the drug.

Primary outcomes include IS, dP/dT max, left ventricular ejection fraction (LVEF), left ventricular developed pressure (LVDP), and left ventricular fractional shortening (LVFS). Secondary outcomes included aortic flow (AF), coronary flow (CF), cardiac troponin T (cTnT), creatine kinase (CK), creatine kinase-MB (CK-MB), lactate dehydrogenase (LDH), tumor necrosis factor-α (TNF-α), interleukin-1β (IL-1β), superoxide dismutase (SOD), malondialdehyde (MDA), and apoptosis rate. Engauge Digitizer was used to extract data from the figures in the included research.

### Quality assessment

Two authors independently assessed the quality of included studies using SYRCLE’s RoB tool, which consists of ten categories (Hooijmans et al., 2014).

### Statistical analysis

The standard mean difference (SMD) was used to calculate the summary statistics and 95% confidence interval (CI) of RES in the primary and secondary outcomes. I^2^ statistics were used for the heterogeneity assessment. Generally, heterogeneity was divided into low (0–25%), moderate (25%–75%), and high (>75%) by I^2^ value. Subgroup analysis was conducted to probe the source of the heterogeneity. When more than eight studies were conducted publication bias analysis by sensitive analysis, funnel plot, Begg’s test, and Egger’s test. Univariate meta-regression was conducted to explore the heterogeneity of IS in MIRI model. The dosage-efficacy analysis was plotted by GraphPad Prism 10.2.0. R (Version 4.2.2) software and its packages ‘meta’ and ‘metafor’ were used to conduct all the analysis above. *P* < 0.05 was considered statistically significant.

## Results

### Studies selection

**Figure 1:**
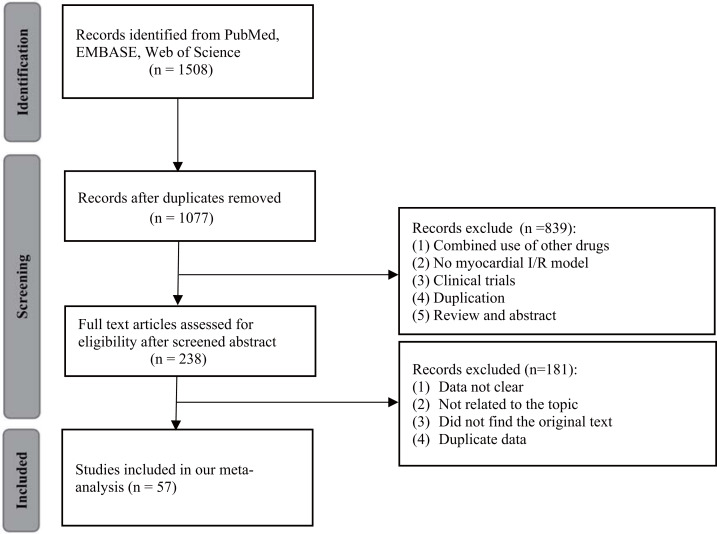
Flow diagram of database searches and study selection.

Figure 1 showed the flow diagram. A total of 1,508 studies were involved in initial searching in PubMed (*n* = 247), Web of Science (*n* = 616), and EMBASE (*n* = 645). Duplicate articles (*n* = 431) were removed by EndNote. After reading the title and abstract, 238 articles were selected. Subsequently, 181 studies were excluded when screening the full text. Ultimately, this meta-analysis included 57 studies with 1,125 animals (Xuan et al., 2012; Adam et al., 2013; Ahmet et al., 2016; Boshra, 2020; Bradamante et al., 2003; Burstein et al., 2007; Cong et al., 2014; Das et al., 2005; Das et al., 2006; Dernek et al., 2004; Dong et al., 2015; Du et al., 2014; Feng et al., 2019; Gu et al., 2014; Hale & Kloner, 2001; He et al., 2021; Hung et al., 2000; Kaga et al., 2005; Kanamori et al., 2013; Kazemirad & Kazerani, 2020; Lamont et al., 2011; Lekli et al., 2010; Lekli et al., 2008; Li, Duan & Shen, 2022; Li et al., 2015; Li et al., 2024; Liao et al., 2015; Lin et al., 2008; Liu et al., 2022; Liu et al., 2019; Liu et al., 2023; Manjunatha et al., 2020; Matsumura et al., 2018; Mei, Liu & Sun, 2021; Mukhopadhyay et al., 2010; Naumenko et al., 2013; Penumathsa et al., 2007; Raj et al., 2016; Raj et al., 2021; Ray et al., 1999; Rogers & Otis, 2017; Salian et al., 2024; Sato, Maulik & Das, 2002; Shalwala et al., 2014; Shen et al., 2006; Shen et al., 2012; Singh et al., 2024; Soltan et al., 2021; Tanno et al., 2010; Thuc et al., 2012; Tian, Xiong & Xia, 2023; Xi et al., 2009a; Xin et al., 2010; Xu et al., 2022; Yang et al., 2016; Zhu et al., 2024; Hung, Su & Chen, 2004).

### Characteristics of included studies

Of the included studies, 34 studies used Sprague-Dawley (SD) rats, eight studies used Wistar rats, one study used Zucker rats, one study used Long-Evans rats, one study used albino rats, seven studies used C57BL/6 mice, two study used Kun-Ming mice, one study used ICR mice, one study used To-2 hamster, and one study used New Zealand White rabbits. To build experimental animal models, 39 articles used MIRI by LAD ligation, ISO injection, or cardioplegia, and 18 articles used chronic MI by permanent LAD ligation. RES treatment was delivered by multiple routes, including perfused heart in 12 articles, oral administration in 25 articles, intraperitoneal injection in 14 articles, intravenous injection in four articles, subcutaneously embedded osmotic minipumps in one article, and injection surrounding the infarcted heart area in one article. The intervention duration varied between 5 min to 10 months. Studies were reported between 1999 and 2024. The details of the basic information about the trials included in the present meta-analysis were shown in Table 1.

**Table 1:** Baseline characteristics of included studies.

**Author**	**Year**	**Country**	**Species**	**n(RES/Ctrl)**	**Anesthetic**	**Model method**	**Drug delivery method (Dose)**	**Duration**	**Outcomes**
**MIRI animal model**									
Adam	2013	South Africa	Long-Evans rats	73/40	Sodium pentobarbitone (60 mg/kg)	Ischemia for 30 min then reflow for 60 min	Perfuse (10 μM)	15 min	1. Infarction size 2. Coronary flow 3. LVDP
Boshra	2020	Egypt	SD rats	10/10	NA	ISO	Oral (50 mg/kg/d)	4 weeks	1. LDH 2. CK-MB 3. cTnT 4. SOD 5. MDA
Bradamante	2003	Netherlands	SD rats	6/6	Sodium pentobarbital	low-flow ischemia (0.6 ml/min) for 30 min then reperfusion for 30 min	Perfuse (10 μM)	10 min	1. Coronary flow
Cong	2014	China	SD rats	10/10	Sodium pentobarbitone (40 mg/kg)	Ischemia for 30 min then reflow for 2 h	Intravenous (100 umol/L)	5 min	1. Infarction size 2. LDH 3. CK 4. TNF-α
Das	2005	America	SD rats	6/6	Sodium pentobarbital (80 mg/kg)	Ischemia for 30 min then reflow for 2 h	Oral (2.5 mg/kg/d)	1 week	1. Infarction size 2. Aortic flow 3. Coronary flow 4. LVDP 5. dP/dt max
Das	2006	America	SD rats	6/6	Sodium pentobarbital (80 mg/kg)	Ischemia for 30 min then reflow for 2 h	Perfuse (10 μM)	15 min	1. Infarction size 2. Aortic flow 3. Coronary flow 4. LVDP 5. dP/dt max
Dernek	2004	Turkey	SD rats	10/10	Sodium pentobarbital (50 mg/kg)	Ischemia for 60 min then reflow for 1H	Oral (20 mg/kg/d)	2 weeks	1. Coronary flow 2. dP/dt max 3. LDH 4. CK-MB 5. cTnT 6. MDA
Dong	2015	China	SD rats	10/10	NR	Ischemia for 30 min then reflow for 2 h	Intraperitoneal (10 mg/kg)	60 min	1. Infarction size 2. CK-MB 3. cTnT 4. IL-1β
Du	2014	China	C57BL/6 mice	8/8	Isoflurane	Ischemia for 30 min then reflow for 24 h	Intraperitoneal (10 mg/kg)	60 min	1. Infarction size 2. LDH 3. Apoptosis rate
Feng	2019	China	SD rats	8/8	NR	ISO	Oral (10 mg/kg)	1 week	1. Infarction size 2. LDH 3. CK-MB 4. SOD 5. MDA 6. Apoptosis rate
Hale	2001	American	New Zealand White rabbits	8/8	Ketamine (200 mg) + xylazine (100 mg)	Ischemia for 30 min then reflow for 3 h	Oral (1.5 mg/kg)	15 min	1. Infarction size
Hung	2000	China	SD rats	6/7	Urethane (1.25 g/kg)	Ischemia for 30 min then reflow for 30 min	Intravenous (23 ug/Kg)	15 min	1. LDH
Hung	2004	China	SD rats	8/8	Urethane (1.25 g/kg)	Ischemia for 60 min then reflow for 3 h	Intraperitoneal (1 mg/kg)	60 min	1. Infarction size 2. LDH 3. CK
Kazemirad	2020	Iran	Wistar rats	10/10	Thiopental sodium (50 mg/kg)	Ischemia for 30 min then reflow for 2 h	Perfuse (10 μM)	30 min	1. Infarction size 2. LVDP 3. dP/dt max 4. LDH 5. CK-MB 6. SOD 7. MDA
Lamont	2011	South Africa	Wistar rats	6/6	Sodium pentobarbitone (60 mg/kg)	Ischemia for 30 min then reflow for 2 h	Perfuse (10 μM)	15 min	1. Infarction size 2. Coronary flow 3. LVDP
Lekli	2008	Hungary	Zucker rats	6/6	Pentobarbital sodium (60 mg/kg)	Ischemia for 30 min then reflow for 2 h	Oral (5 mg/kg/d)	2 weeks	1. Infarction size 2. Aortic flow 3. Coronary flow 4. LVDP
Lekli	2010	America	SD rats	3/3	Sodium pentobarbital (80 mg/kg)	Ischemia for 30 min then reflow for 2 h	Oral (2.5 mg/kg/d)	2 weeks	1. Infarction size 2. Aortic flow 3. Coronary flow 4. LVDP 5. dP/dt max
Li	2022	China	SD rats	18/18	Sodium pentobarbital (40 mg/kg)	Ischemia for 30 min then reflow for 2 h	Intraperitoneal (50 mg/kg/d)	2 weeks	1. Infarction size 2. Apoptosis rate
Li	2015	China	SD rats	10/10	Sodium pentobarbital (40 mg/kg)	Ischemia for 30 min then reflow for 2 h	Intravenous (100 uM)	5 min	1. Infarction size 2. TNF-α 3. Apoptosis rate
Liao	2015	China	Kun-Ming mice	6/6	Sodium pentobarbital (30 mg/kg)	Ischemia for 30 min then reflow for 30 min	Oral (2 mg/kg/d)	6 weeks	1. Infarction size 2. LDH 3. CK
Li	2024	China	Kun-Ming mice	6/6	NR	Ischemia for 30 min then reflow for 24 h	Intraperitoneal (25 mg/kg/d)	4 weeks	1. Infarction size 2. Apotosis index
Liu	2023	China	SD rats	8/8	Isoflurane	Ischemia for 30 min then reflow for 2 h	Oral (30 mg/kg/d)	1 week	1. Infarction size 2. LDH 3. CK-MB 4. LVEF 5. LVFS
Manjunatha	2020	India	Wistar rats	6/6	NR	ISO	Oral (50 mg/kg/d)	4 weeks	1. LDH 2. CK-MB
Mukhopadhyay	2010	America	SD rats	6/6	Sodium pentobarbital (80 mg/kg)	Ischemia for 30 min then reflow for 2 h	Oral (5 mg/kg/d)	3 weeks	1. Infarction size 2. Aortic flow 3. LVDP 4. dP/dt max 5. Apoptosis rate
Naumenko	2013	America	Wistar rats	9/10	Urethane (2.0 mg/kg)	Ischemia for 30 min then reflow for 2 h	Intraperitoneal (10 mg/kg)	30 min	1. Infarction size
Penumathsa	2007	Japan	SD rats	6/6	Ketamine (100 mg/kg) + xylazine (10 mg/kg)	Ischemia for 30 min then reflow for 2 h	Oral (20 mg/kg/d)	2 weeks	1. Infarction size 2. dP/dt max
Ray	1999	Italy	SD rats	9/9	Sodium pentobarbital (80 mg/kg)	Ischemia for 30 min then reflow for 2 h	Perfuse (10 μM)	15 min	1. Infarction size 2. Aortic flow 3. Coronary flow 4. dP/dt max 5. MDA
Sato	2002	America	SD rats	6/6	Sodium pentobarbital (80 mg/kg)	Ischemia for 30 min then reflow for 2 h	Perfuse (10 μM)	15 min	1. Infarction size 2. Apoptosis rate
Shalwala	2014	China	ICR mice	6/6	Sodium pentobarbital (70 mg/kg)	Ischemia for 30 min then reflow for 24 h	Intraperitoneal (5 mg/kg)	1 day	1. Infarction size
Shen	2006	China	SD rats	8/8	Sodium pentobarbital (45 mg/kg)	Ischemia for 30 min then reflow for 2 h	Intravenous (10 uM)	2 weeks	1. Infarction size 2. dP/dt max 3. MDA
Shen	2012	China	SD rats	10/10	Pentobarbital sodium (45 mg/kg)	Ischemia for 2 h then reflow for 4 h	Perfuse (100 μM)	15 min	1. LDH 2. SOD 3. MDA
Salian	2024	India	Albino rats	6/6	NR	ISO	Oral (20 mg/kg/d)	4 weeks	1. CK-MB 2. LDH 3. SOD (U/L)
Singh	2024	India	Wistar rats	6/6	Ketamine (80 mg/kg) + xylazine (10 mg/kg)	Ischemia for 45 min then reflow for 60 min with (high fat diet for 10 weeks before MIRI)	Oral (20 mg/kg/d)	10 weeks	1. Infarction size 2. IL-1β, 3. IL-6 4. TNF-α 5. LDH 6. CK-MB 7. SOD
Soltan	2021	Iran	Wistar rats	6/6	Ketamine (70 mg/kg) + xylazine (10 mg/kg)	Ischemia for 30 min then reflow for 2 h	Oral (2.5 mg/kg/d)	2 weeks	1. Infarction size
Thuc	2012	Japan	SD rats	5/5	Ketamine (60 mg/kg) + xylazine (10 mg/kg)	Ischemia for 20 min then reflow for 30 min	Perfuse (20 μM)	15 min	1. Infarction size 2. LVDP
Tian	2023	China	C57BL/6 mice	6/6	Sodium pentobarbital (60 mg/kg)	Ischemia for 30 min then reflow for 2 h	Intraperitoneal (30 mg/kg/d)	1 week	1. Infarction size 2. LVEF 3. LVFS 4. LDH 5. CK-MB
Xi	2009	China	Wistar rats	6/6	Thiobutabarbital sodium (100 mg/kg)	Ischemia for 30 min then reflow for 2 h	Perfuse (10 μM)	5 min	1. Infarction size
Xu	2022	China	SD rats (T2DM)	10/10	NR	Ischemia for 30 min then reflow for 2 h	Intraperitoneal (20 mg/kg/d)	1 week	1. Infarction size 2. CK-MB 3. SOD
Yang	2016	China	SD rats	10/10	Chloral hydrate	Ischemia for 30 min then reflow for 1 h	Perfuse (10 μM)	15 min	1. Infarction size 2. Apoptosis rate
**Chronic MI model**									
Ahmet	2016	America	Wistar rats	9/8	Isoflurane (2% in oxygen)	LAD ligation	Oral (5 mg/kg/d)	10 months	1. Infarction size 2. LVEF 3. dP/dt max
Burstein	2007	Canada	SD rats	24/26	Sodium pentobarbital	LAD ligation	Oral (17 mg/kg/d)	12 weeks	1. LVFS 2. dP/dt max
Gu	2014	China	SD rats	9/4	Ketamine hydrochloride (80 mg/kg) + xylazine (12 mg/kg)	LAD ligation	Oral (2.5 mg/kg)	16 weeks	1. LVEF
He	2021	China	C57BL/6 mice	50/50	Sodium pentobarbital (80 mg/kg)	LAD ligation	Intraperitoneal (2 mg/kg)	4 weeks	1. Infarction size 2. SOD 3. Apoptosis rate
Kaga	2005	Japan	SD rats	6/6	Ketamine (100 mg/kg) + xylazine (10 mg/kg)	LAD ligation	Oral (1 mg/kg/d)	2 weeks	1. Infarction size
Kanamori	2013	Japan	C57BL/6 mice	16/16	Urethane (1.25 g/kg)	LAD ligation	Subcutaneously embedded osmotic minipumps (50 mg/kg/d)	4 weeks	1. Infarction size 2. LVEF 3. LVDP
Lin	2008	China	SD rats	12/11	Isoflurane	LAD ligation	Intraperitoneal (1mg/kg/d)	4 weeks	1. Infarction size 2. LVFS 3. LVDP
Liu	2019	China	C57BL/6 mice	12/12	Sodium pentobarbital (50 mg/kg)	LAD ligation	Oral (20 mg/kg/d)	4 weeks	1. LVEF 2. LVFS 3. IL-1β 4. TNF-α
Liu	2022	China	SD rats	5/5	Ketamine (100 mg/kg) + xylazine (10 mg/kg)	LAD ligation	Resveratrol (20 mg/d) inject surrounding the infarcted heart area	1 week	1. IL-1β 2. MDA
Matsumura	2018	Canada	SD rats	6/7	Isoflurane	LAD ligation	Oral (0.2 g/kg in diet)	2 weeks	1. LVEF
Mei	2021	China	SD rats	8/8	Sodium pentobarbital	LAD ligation	Intraperitoneal (8 mg/kg)	4 weeks	1. LVEF 2. LVFS
Raj	2021	Canada	SD rats	6/6	Isoflurane	LAD ligation	Oral (2.5 mg/kg/d)	8 weeks	1. Infarction size 2. LVEF 3. TNF-α 4. MDA
Raj	2016	Canada	SD rats	9/13	Isoflurane	LAD ligation	Oral (2.5 mg/kg/d)	8 weeks	1. LVEF
Rogers	2017	America	SD rats	6/6	Isoflurane	LAD ligation	Intraperitoneal (10 mg/kg/d)	3 weeks	1. Infarction size 2. Apoptosis rate
Tanno	2010	Japan	TO-2 hamster	15/15	NR	LAD ligation	Oral (4 g/kg in diet)	6 weeks	1. LVEF 2. LVFS
Xin	2010	China	SD rats	6/6	Sodium pentobarbital (50 mg/kg)	LAD ligation	Intraperitoneal (1 mg/kg/d)	4 weeks	1. Infarction size 2. IL-1β 3. SOD 4. MDA
Xuan	2012	China	C57BL/6 mice	8/8	Ketamine (100 mg/ kg) + xylazine (5 mg/kg)	LAD ligation	Intraperitoneal (20 mg/kg/d)	6 weeks	1. Infarction size 2. LVFS
Zhu	2024	China	C57BL/6J mice	8/8	Sodium pentobarbital (50 mg/kg)	LAD ligation	Oral (45.5 mg/kg/d)	7 weeks	1. Infarction size 2. LVEF 3. LVFS

**Notes.**

SD rats Sprague-Dawley rats ISO isoproterenol LAD left anterior branch LVEF left ventricular ejection fraction LVFS left ventricular fractional shortening dP/dT max maximum 1st derivative of developed pressure LVDP left ventricular developed pressure CK creatine kinase CK-MB creatine kinase isoenzyme LDH lactic dehydrogenase cTnT cardiac troponin T SOD superoxide dismutase MDA malondialdehyde IL-1β interleukin-1β TNF-α tumor necrosis factor-α NR not reported

(Xuan et al., 2012; Adam et al., 2013; Ahmet et al., 2016; Boshra, 2020; Bradamante et al., 2003; Burstein et al., 2007; Cong et al., 2014; Das et al., 2005; Das et al., 2006; Dernek et al., 2004; Dong et al., 2015; Du et al., 2014; Feng et al., 2019; Gu et al., 2014; Hale & Kloner, 2001; He et al., 2021; Hung et al., 2000; Kaga et al., 2005; Kanamori et al., 2013; Kazemirad & Kazerani, 2020; Lamont et al., 2011; Lekli et al., 2010; Lekli et al., 2008; Li, Duan & Shen, 2022; Li et al., 2015; Li et al., 2024; Liao et al., 2015; Lin et al., 2008; Liu et al., 2022; Liu et al., 2019; Liu et al., 2023; Manjunatha et al., 2020; Matsumura et al., 2018; Mei, Liu & Sun, 2021; Mukhopadhyay et al., 2010; Naumenko et al., 2013; Penumathsa et al., 2007; Raj et al., 2016; Raj et al., 2021; Ray et al., 1999; Rogers & Otis, 2017; Salian et al., 2024; Sato, Maulik & Das, 2002; Shalwala et al., 2014; Shen et al., 2006; Shen et al., 2012; Singh et al., 2024; Soltan et al., 2021; Tanno et al., 2010; Thuc et al., 2012; Tian, Xiong & Xia, 2023; Xi et al., 2009a; Xin et al., 2010; Xu et al., 2022; Yang et al., 2016; Zhu et al., 2024; Hung, Su & Chen, 2004).

The methodological quality scores of the included literature ranged from 4 to 7 (Table 2). The potential molecular mechanism of cardioprotective effect of RES from MIRI and MI was summarized in Table 3.

**Table 2:** Quality assessment of included studies.

**Author**	**Year**	**A**	**B**	**C**	**D**	**E**	**F**	**G**	**H**	**I**	**J**	**Total**
**MIRI animal model**												
Adam	2013		★	★			★		★	★	★	6
Boshra	2020		★	★			★		★	★	★	6
Bradamante	2003		★	★			★			★		4
Cong	2014		★	★			★		★	★		5
Das	2006		★	★			★		★	★		5
Das	2005		★	★			★		★	★	★	6
Dernek	2004		★	★			★		★	★	★	6
Dong	2015		★	★			★		★	★	★	6
Du	2014		★	★			★		★	★	★	6
Feng	2019		★	★			★		★	★		5
Hale	2001		★	★			★			★		4
Hung	2000		★	★			★		★	★	★	6
Hung	2004		★	★			★		★	★	★	6
Kazemirad	2020		★	★			★		★	★	★	6
Lamont	2011		★	★			★		★	★	★	6
Lekli	2010		★	★			★		★	★	★	6
Lekli	2008		★	★			★		★	★	★	6
Li	2022		★	★			★		★	★	★	6
Li	2015		★	★			★		★	★	★	6
Li	2024		★	★			★		★	★	★	6
Liao	2015		★	★	★		★		★	★	★	6
Liu	2023		★	★			★		★	★	★	7
Manjunatha	2020		★	★			★		★	★	★	6
Mukhopadhyay	2010		★	★			★		★	★	★	6
Naumenko	2013		★	★			★			★		4
Penumathsa	2007		★	★			★		★	★	★	6
Ray	1999		★	★			★		★	★		5
Salian	2024		★	★					★	★	★	5
Sato	2002		★	★			★		★	★		5
Shalwala	2014		★	★		★	★		★	★	★	7
Shen	2006		★	★			★		★	★	★	6
Shen	2012		★	★			★		★			4
Singh	2024		★	★			★		★	★	★	6
Soltan	2021		★	★			★		★		★	5
Thuc	2012		★	★			★		★	★	★	6
Tian	2023		★	★			★		★	★	★	6
Xi	2009		★	★			★		★	★	★	6
Xu	2022		★	★			★		★	★		5
Yang	2016		★	★			★		★	★	★	6
**Chronic MI model**												
Ahmet	2016		★	★			★		★	★	★	6
Burstein	2007		★	★			★		★	★	★	6
Gu	2014		★	★			★		★	★	★	6
He	2021		★	★			★		★	★	★	6
Kaga	2005		★	★			★		★	★	★	6
Kanamori	2013		★	★			★		★	★	★	6
Lin	2008		★	★			★		★	★		5
Liu	2019		★	★			★		★	★	★	6
Liu	2022		★	★			★		★	★	★	6
Matsumura	2018		★	★			★		★	★	★	6
Mei	2021		★	★			★		★	★		5
Raj	2021		★	★			★		★			4
Raj	2016		★	★			★		★	★	★	6
Rogers	2017		★	★			★		★	★	★	6
Tanno	2010		★	★			★		★	★	★	6
Xin	2010		★	★			★		★	★	★	6
Xuan	2012		★	★			★		★	★	★	6
Zhu	2024		★	★	★		★		★	★	★	7

**Notes.**

A Sequence generation B Baseline characteristics C Allocation concealment D Random housing E Blinding investigators F Random outcome assessment G Blinding outcome assessor H Incomplete outcome data I Selective outcome reporting J Other sources of bias

(Xuan et al., 2012; Adam et al., 2013; Ahmet et al., 2016; Boshra, 2020; Bradamante et al., 2003; Burstein et al., 2007; Cong et al., 2014; Das et al., 2005; Das et al., 2006; Dernek et al., 2004; Dong et al., 2015; Du et al., 2014; Feng et al., 2019; Gu et al., 2014; Hale & Kloner, 2001; He et al., 2021; Hung et al., 2000; Kaga et al., 2005; Kanamori et al., 2013; Kazemirad & Kazerani, 2020; Lamont et al., 2011; Lekli et al., 2010; Lekli et al., 2008; Li, Duan & Shen, 2022; Li et al., 2015; Li et al., 2024; Liao et al., 2015; Lin et al., 2008; Liu et al., 2022; Liu et al., 2019; Liu et al., 2023; Manjunatha et al., 2020; Matsumura et al., 2018; Mei, Liu & Sun, 2021; Mukhopadhyay et al., 2010; Naumenko et al., 2013; Penumathsa et al., 2007; Raj et al., 2016; Raj et al., 2021; Ray et al., 1999; Rogers & Otis, 2017; Salian et al., 2024; Sato, Maulik & Das, 2002; Shalwala et al., 2014; Shen et al., 2006; Shen et al., 2012; Singh et al., 2024; Soltan et al., 2021; Tanno et al., 2010; Thuc et al., 2012; Tian, Xiong & Xia, 2023; Xi et al., 2009a; Xin et al., 2010; Xu et al., 2022; Yang et al., 2016; Zhu et al., 2024; Hung, Su & Chen, 2004).

**Table 3:** The molecular and cellular mechanisms underlying the cardio-protection effect of resveratrol treatment in MIRI and MI.

Author	Year	Proposed mechanisms
**MIRI animal model**		
Adam	2013	Activate Sirt1
Boshra	2020	Increase the antioxidant, antiapoptotic, and anti-inflammatory capacities
Bradamante	2003	NA
Cong	2014	Increase in NO production, the inhibition of neutrophil accumulation, TNF-α induction and cGMP signaling pathways in myocardium
Das	2005	potentiates the expression of KDR, which in turn upregulates eNOS expression
Das	2006	Anti-inflammatory action through NO-dependent mechanism
Dernek	2004	Free radical scavenging and antioxidant
Dong	2015	Inhibite the expression and activation of NALP3 inflammasome pathway
Du	2014	Resveratrol exerts cardioprotective effects through AMPK -Kir6.2/K-ATP signal pathway
Feng	2019	Resveratrol exerts cardioprotective effects through VEGF-B/AMPK/eNOS/NO signal pathway
Hale	2001	NA
Hung	2000	Antioxidant and upregulate NO production
Hung	2004	Anti-oxidant activity and upregulate NO production
Kazemirad	2020	Anti-oxidant activity mediated *via* NO signaling
Lamont	2011	Activate survivor activating factor enhancement (SAFE) pathway involves the activation of TNF-α and STAT3
Lekli	2008	Increase GLUT-4 expression, reduce endothelin expression, and reduce cardiac apoptosis
Lekli	2010	Acticate Akt-Bcl-2 survival pathway and enhanced autophagy
Li	2022	Inhibit the expression of STIM2 by promoting the expression of miR-20b-5p
Li	2015	Inhibit TLR4/NF-kB signaling and anti-inflammatory
Li	2024	Inhibit AKT nitration modification
Liao	2015	Down-regulates VDAC1, leading to prevention of mPTP opening and cardiomyocyte apoptosis
Liu	2023	Inhibit autophagy by DJ-1/MEKK1/JNK pathway
Manjunatha	2020	Inhibit NF-kB and TNF-α signalling pathways
Mukhopadhyay	2010	Regulate miRNA expression
Naumenko	2013	NA
Penumathsa	2007	Pro-angiogenic and anti-apoptotic effects
Ray	1999	Scavenge peroxyl radical
Salian	2024	NA
Sato	2002	Anti-oxidative stress by HSP70 and anti-death signal by JNK-1 and c-Jun
Shalwala	2014	Activate Sirt1
Shen	2006	Antioxidant and upregulation of NO production
Shen	2012	Prevent cell apoptosis, decreasing LDH release and increasing ATPase activity.
Singh	2024	Target TLR4/MyD88/NF-κB/iNOS signaling cascade
Soltan	2021	Enhancement of Ang (1-7)/MasR axis
Thuc	2012	Reduce ROS and preserving mitochondrial function
Tian	2023	Suppressing the activation of Hippo pathway and increasing the nuclear translocation of YAP
Xi	2009	Inactivates GSK-3β and induces GSK-3β translocation to mitochondria through the cGMP/PKG signaling pathway
Xu	2022	Inhibit oxidative stress and alleviate MIRI by activating the AMPK/p38/ Nrf2 signaling pathway
Yang	2016	Promotion of VEGF-B/ antioxidant signaling pathway
**Chronic MI model**		
Ahmet	2016	NA
Burstein	2007	Increase adrenergic receptor binding density
Gu	2014	Activating SIRT1-dependent transcriptional regulatory mechanisms, thereby restoring AMPK expression
He	2021	Inhibit NF- κB- dependent inflammation and enhance antioxidant capacity
Kaga	2005	Mediate cardioprotection and neovascularization through Trx-1-HO-1-VEGF pathway
Kanamori	2013	Increase Autophagy-Activating AMP Kinase Pathway
Lin	2008	Reduction of atrial natriuretic peptide and transforming growth factor-β1
Liu	2019	Promote anti-inflammatory M2-like polarization of macrophages and regulate JAK2-SATA3 phosphorylation
Liu	2022	Inhibit ferroptosis *via* inducing KAT5/GPX4
Matsumura	2018	Inhibit CYP1B1 and cardiotoxic HETEs metabolites
Mei	2021	Regulate the silent information regulator 1/protein kinase R-like endoplasmic reticulum kinase pathway Endoplasmic reticulum stress pathway
Raj	2021	Reduction in cardiac oxidative stress, inflammation and fibrosis
Raj	2016	Reduce oxidative stress by preventing the decrease in the activity of superoxide dismutase and catalase, and decrease cardiac inflammation and fibrosis
Rogers	2017	Induces KLF15 expression
Tanno	2010	Induce Mn-SOD *via* nuclear SIRT1 reduced oxidative stress
Xin	2010	Attenuation of oxidative stress and inflammatory response, inhibition of NGF expression, and protection against of sympathetic neural remodeling
Xuan	2012	Down-regulate of fractalkine (FKN)
Zhu	2024	Activa SIRT3/FOXO3a signaling pathway and restore redox homeostasis

**Notes.**

(Xuan et al., 2012; Adam et al., 2013; Ahmet et al., 2016; Boshra, 2020; Bradamante et al., 2003; Burstein et al., 2007; Cong et al., 2014; Das et al., 2005; Das et al., 2006; Dernek et al., 2004; Dong et al., 2015; Du et al., 2014; Feng et al., 2019; Gu et al., 2014; Hale & Kloner, 2001; He et al., 2021; Hung et al., 2000; Kaga et al., 2005; Kanamori et al., 2013; Kazemirad & Kazerani, 2020; Lamont et al., 2011; Lekli et al., 2010; Lekli et al., 2008; Li, Duan & Shen, 2022; Li et al., 2015; Li et al., 2024; Liao et al., 2015; Lin et al., 2008; Liu et al., 2022; Liu et al., 2019; Liu et al., 2023; Manjunatha et al., 2020; Matsumura et al., 2018; Mei, Liu & Sun, 2021; Mukhopadhyay et al., 2010; Naumenko et al., 2013; Penumathsa et al., 2007; Raj et al., 2016; Raj et al., 2021; Ray et al., 1999; Rogers & Otis, 2017; Salian et al., 2024; Sato, Maulik & Das, 2002; Shalwala et al., 2014; Shen et al., 2006; Shen et al., 2012; Singh et al., 2024; Soltan et al., 2021; Tanno et al., 2010; Thuc et al., 2012; Tian, Xiong & Xia, 2023; Xi et al., 2009a; Xin et al., 2010; Xu et al., 2022; Yang et al., 2016; Zhu et al., 2024; Hung, Su & Chen, 2004).

### Infarct size

**Figure 2:**
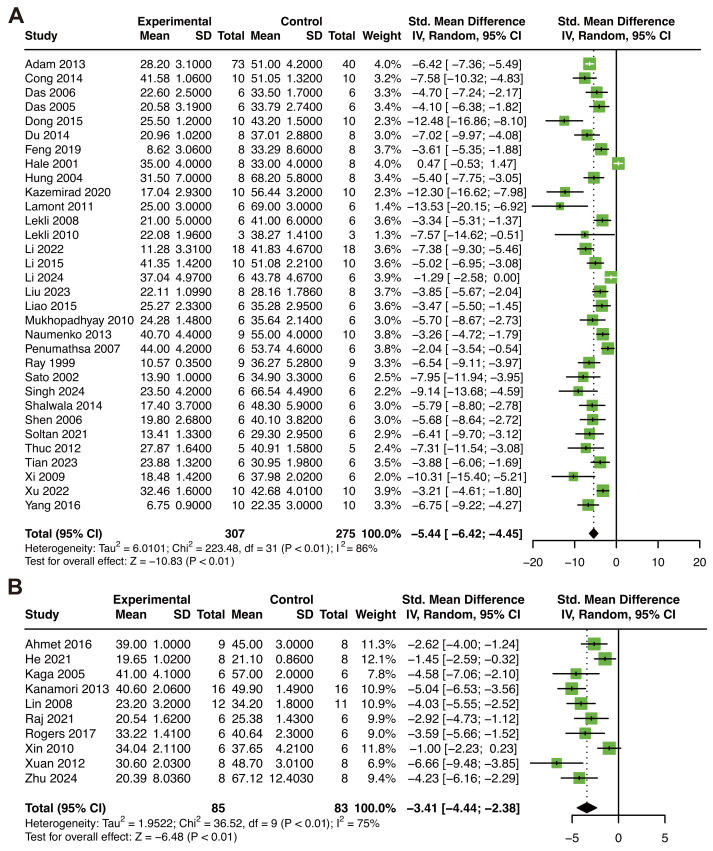
Forest plot showing cardioprotective effects of resveratrol on infarction size in animal models of MIRI (A) and MI (B). MIRI, myocardial ischemia/reperfusion injury; MI, myocardial infarction. (Xuan et al., 2012; Adam et al., 2013; Ahmet et al., 2016; Boshra, 2020; Bradamante et al., 2003; Burstein et al., 2007; Cong et al., 2014; Das et al., 2005; Das et al., 2006; Dernek et al., 2004; Dong et al., 2015; Du et al., 2014; Feng et al., 2019; Gu et al., 2014; Hale & Kloner, 2001; He et al., 2021; Hung et al., 2000; Kaga et al., 2005; Kanamori et al., 2013; Kazemirad & Kazerani, 2020; Lamont et al., 2011; Lekli et al., 2010; Lekli et al., 2008; Li, Duan & Shen, 2022; Li et al., 2015; Li et al., 2024; Liao et al., 2015; Lin et al., 2008; Liu et al., 2022; Liu et al., 2019; Liu et al., 2023; Manjunatha et al., 2020; Matsumura et al., 2018; Mei, Liu & Sun, 2021; Mukhopadhyay et al., 2010; Naumenko et al., 2013; Penumathsa et al., 2007; Raj et al., 2016; Raj et al., 2021; Ray et al., 1999; Rogers & Otis, 2017; Salian et al., 2024; Sato, Maulik & Das, 2002; Shalwala et al., 2014; Shen et al., 2006; Shen et al., 2012; Singh et al., 2024; Soltan et al., 2021; Tanno et al., 2010; Thuc et al., 2012; Tian, Xiong & Xia, 2023; Xi et al., 2009a; Xin et al., 2010; Xu et al., 2022; Yang et al., 2016; Zhu et al., 2024; Hung, Su & Chen, 2004).

The meta-analysis of IS in animal models of MIRI included 32 publications with 582 animals (Adam et al., 2013; Cong et al., 2014; Das et al., 2005; Das et al., 2006; Dong et al., 2015; Du et al., 2014; Feng et al., 2019; Hale & Kloner, 2001; Hung et al., 2000; Kazemirad & Kazerani, 2020; Lamont et al., 2011; Lekli et al., 2010; Lekli et al., 2008; Li, Duan & Shen, 2022; Li et al., 2015; Li et al., 2024; Liao et al., 2015; Liu et al., 2023; Mukhopadhyay et al., 2010; Naumenko et al., 2013; Penumathsa et al., 2007; Ray et al., 1999; Sato, Maulik & Das, 2002; Shalwala et al., 2014; Shen et al., 2006; Singh et al., 2024; Soltan et al., 2021; Thuc et al., 2012; Tian, Xiong & Xia, 2023; Xi et al., 2009a; Xu et al., 2022; Yang et al., 2016). RES supplementation was correlated with a decline of IS (SMD: −5.44; 95% CI [−6.42 to −4.45]; *P* < 0.01; I^2^ = 86%; Fig. 2A). Subgroup analysis based on country, IS measure methods, ischemia time, and duration did not show significant differences in heterogeneity between groups (Table 4). However, the drug delivery methods, reperfusion time of RES, and species showed a significant difference between subgroups. RES delivery by perfusion and intravenous, ISO method, and the mice group showed a decrease of heterogeneity within subgroups (Table 4). Sensitivity analysis revealed that our result was stable (Fig. S1A). The funnel plot was asymmetry, Begg’s test (*P* < 0.01), and Egger’s test (*P* < 0.01) of MIRI models showed the existence of publication bias (Fig. S1C).

Moreover, 10 researches (168 animals) were included in the analysis of IS in the animal model of MI (Xuan et al., 2012; Ahmet et al., 2016; He et al., 2021; Kaga et al., 2005; Kanamori et al., 2013; Lin et al., 2008; Raj et al., 2021; Rogers & Otis, 2017; Xin et al., 2010; Zhu et al., 2024). The RES treatment decreased IS (SMD: −3.41; 95% CI [−4.44 to −2.38]; *P* < 0.01; I^2^ = 75%; Fig. 2B). Subgroup analysis of species and drug delivery did not reveal significant differences in heterogeneity between groups (Table 5). In the subgroup of dose, the group of 1–5 mg/kg and >5 mg/kg presented a decrease in heterogeneity within subgroups. Besides, the group of >5 mg/kg was the lowest SMD, which means that the dose >5 mg/kg may have a better therapeutic effect in the animal model of MI. Sensitivity analysis revealed that our result was stable (Fig. S1B). The funnel plot was symmetry, Begg’s test (*P* = 0.10) and Egger’s test (*P* = 0.06) of MI models showed the absence of publication bias (Fig. S1D).

**Table 4:** Subgroup analysis of pooled estimates of infarct size in MIRI models of MIRI.

Subgroup	No. of studies	SMD	95% CI	*P* value between subgroups	Heterogeneity within subgroups	
					I^2^ (%)	*P* value
**Country**				0.48		
China	16	−5.33	−6.52, −4.15		78	<0.01
America	7	−4.10	−6.28, −1.91		88	<0.01
Japan	2	−4.29	−9.40, 0.82		81	0.02
South Africa	2	−9.19	−15.99, −2.40		77	0.04
Iran	2	−9.18	−14.94, −3.42		78	0.03
Others	3	−5.88	−9.04, −2.72		73	0.03
**IS measure methods**				0.05		
IS/AAR	20	−5.12	−6.06, −4.19		64	<0.01
IS/LV	9	−5.48	−8.30, −2.67		91	<0.01
NR	3	−6.72	−7.60, −5.84		0	0.38
**Ischemia time**				0.06		
≤30 min	28	−5.53	−6.64, −4.42		88	<0.01
30–60 min	2	−6.74	−10.25, −3.22		51	0.15
ISO	2	−3.73	−4.98, −2.47		0	0.85
**Reperfusion time**				0.03		
≤1 h	5	−6.14	−7.81, −4.47		57	0.06
1–2 h	20	−5.97	−7.21, −4.72		75	<0.01
>3 h	5	−3.58	−6.42, −0.69		92	<0.01
ISO	2	−3.73	−4.98, −2.47		0	0.85
**Drug delivery**				<0.01		
Perfusion	9	−7.40	−8.83, −5.98		49	0.05
Oral	11	−3.79	−5.22, −2.36		84	<0.01
Intravenous	3	−5.88	−7.42, −4.33		11	0.33
Intraperitoneal	9	−5.15	−7.02, −3.28		85	<0.01
**Duration**				0.13		
≤30 min	13	−6.42	−8.32, −4.52		92	<0.01
30 min–1 day	5	−6.06	−9.45, −2.67		89	<0.01
>1 day	14	−4.39	−5.32, −3.47		58	0.003
**Species**				<0.01		
Rats	26	−5.90	−6.87, −4.93		74	<0.01
Mice	5	−4.01	−5.99, −2.04		78	0.001
Rabbits	1	0.47	−0.53, 1.47		/	/

**Notes.**

SMD standard mean difference IS infarction size AAR area at risk LV left ventricle NR not reported ISO isoprenaline MIRI myocardial ischemia/reperfusion injury

**Table 5:** Subgroup analysis of pooled estimates of infarct size in animal models of MI.

Subgroup	No. of studies	SMD	95% CI	*P* value between subgroups	Heterogeneity within subgroups	
					I^2^ (%)	*P* value
**Species**				0.33		
Rats	6	−2.95	−4.03, −1.87		62	0.02
Mice	4	−4.15	−6.30, −2.00		86	<0.01
**Drug delivery**				0.11		
Oral	3	−3.30	−4.21, −2.38		0	0.39
Intraperitoneal	5	−3.11	−4.96, −1.26		82	<0.01
Subcutaneous	1	−5.04	−6.53, −3.56		/	/
**Dose**				0.001		
≤1 mg/kg	3	−3.06	−5.32, −0.80		84	<0.01
1–5 mg/kg	3	−2.17	−3.12, −1.23		23	0.27
>5 mg/kg	4	−4.72	−5.68, −3.75		12	0.33

**Notes.**

SMD standard mean difference MI myocardial infarction

### Cardiac function

Due to the different research designs of MIRI and MI, different indicators of cardiac function were generally used. LVFS, LVEF, LVDP, dP/dT max, AF, and CF were used as indicators in animal models of MIRI. On the contrary, LVEF and LVFS were used as indicators in animal models of MI.

**Figure 3:**
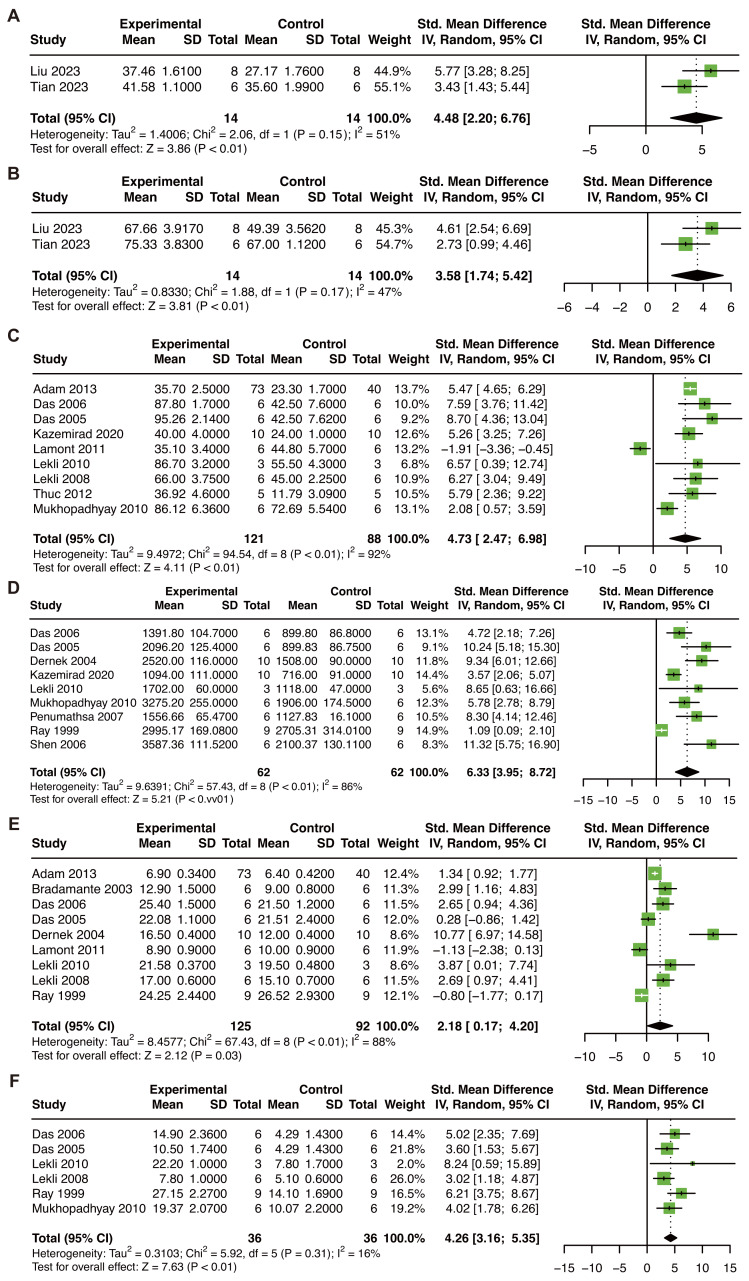
The pooled estimate of LVFS (A), LVEF (B), LVDP (C), dP/dt max (D), CF (E), and AF (F) in MIRI animal model. LVFS, left ventricular fractional shortening; LVEF, left ventricular ejection fraction; LVDP, left ventricular developing pressure; dP/dT max, maximum 1st derivative of developed pressure; AF, aortic flow; CF, coronary flow; MIRI, myocardial ischemia/reperfusion injury. (Liu et al., 2023; Tian, Xiong & Xia, 2023; Adam et al., 2013; Das et al., 2005; Das et al., 2006; Kazemirad & Kazerani, 2020; Lamont et al., 2011; Lekli et al., 2008; Thuc et al., 2012; Mukhopadhyay et al., 2010; Dernek et al., 2004; Lekli et al., 2010; Penumathsa et al., 2007; Ray et al., 1999; Shen et al., 2006; Bradamante et al., 2003).

In animal models of MIRI, the following studies were included for cardiac function analysis: two studies (28 animals) for LVFS and LVEF (Liu et al., 2023; Tian, Xiong & Xia, 2023), nine studies (209 animals) for LVDP (Adam et al., 2013; Das et al., 2005; Das et al., 2006; Kazemirad & Kazerani, 2020; Lamont et al., 2011; Lekli et al., 2010; Lekli et al., 2008; Mukhopadhyay et al., 2010; Thuc et al., 2012), nine studies (124 animals) for dP/dt max (Das et al., 2005; Das et al., 2006; Dernek et al., 2004; Kazemirad & Kazerani, 2020; Lekli et al., 2010; Mukhopadhyay et al., 2010; Penumathsa et al., 2007; Ray et al., 1999; Shen et al., 2006), nine studies (217 animals) for CF (Adam et al., 2013; Bradamante et al., 2003; Das et al., 2005; Das et al., 2006; Dernek et al., 2004; Lamont et al., 2011; Lekli et al., 2010; Lekli et al., 2008; Ray et al., 1999), and six studies (72 animals) for AF (Das et al., 2005; Das et al., 2006; Lekli et al., 2010; Lekli et al., 2008; Mukhopadhyay et al., 2010; Ray et al., 1999). Notably, the LVFS result was derived from two studies, which should be interpreted as exploratory. The results showed that RES treatment significant increase LVFS (SMD: 4.48, *P* < 0.01, I^2^ = 51%; Fig. 3A), LVEF (SMD: 3.58, *P* < 0.01, I^2^ = 47%; Fig. 3B), LVDP (SMD: 4.73, *P* < 0.01, I^2^ = 92%; Fig. 3C), dP/dt max (SMD: 6.33, *P* < 0.01, I^2^ = 86%; Fig. 3D), CF (SMD: 2.18, *P* = 0.03, I^2^ = 88%; Fig. 3E), AF (SMD: 4.26, *P* < 0.01, I^2^ = 16%; Fig. 3F) in animals models of MIRI. The sensitivity analysis of LVDP (Fig. S2A), CF (Fig. S2B), and dP/dt max (Fig. S2C) indicated that our results were stable. The result of the funnel plot, Begg’s test, and Egger’s test in LVDP (Fig. S2D) and CF (Fig. S2E) showed the absence of publication bias. However, publication bias was included in the analysis of dP/dt max (Fig. S2F).

**Figure 4:**
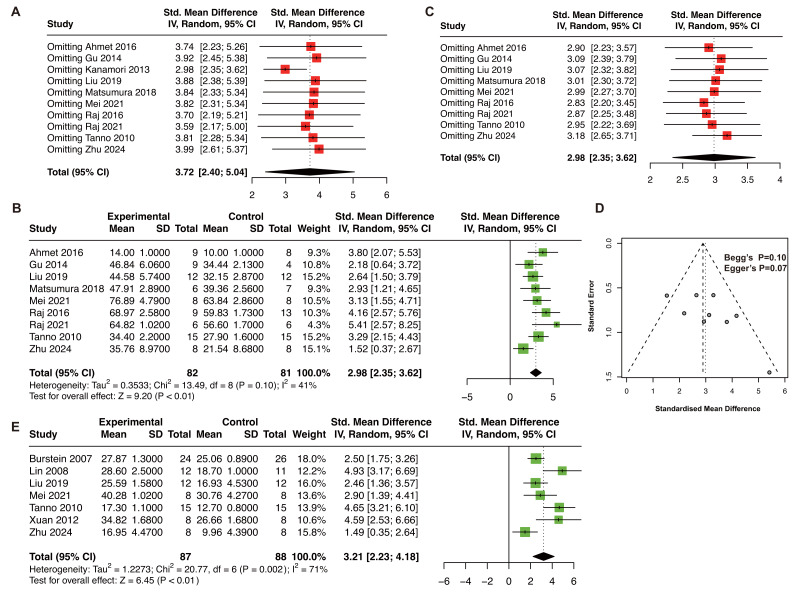
(A) Sensitivity analysis of LVEF in animal models of MI before adjustment. (B) The pooled estimate of LVEF in MI animal model. (C) Sensitivity analysis of LVEF in animal model of MI after adjustment. (D) Funnel plot, Begg’s test, and Egger’s test of LVEF in animal model of MI. LVEF, left ventricular ejection fraction; MI, myocardial infarction. (Ahmet et al., 2016; Gu et al., 2014; Liu et al., 2019; Matsumura et al., 2018; Mei, Liu & Sun, 2021; Raj et al., 2016; Raj et al., 2021; Tanno et al., 2010; Burstein et al., 2007; Lin et al., 2008; Xuan et al., 2012; Zhu et al., 2024).

In the animal model of MI, the pooled estimate of LVEF was 3.72 (*P* < 0.01; I^2^ = 78%). One study was excluded after the sensitivity analysis of LVEF (Fig. 4A) (Kanamori et al., 2013). Finally, nine studies (163 animals) were included in the meta-analysis of LVEF (Ahmet et al., 2016; Gu et al., 2014; Liu et al., 2019; Matsumura et al., 2018; Mei, Liu & Sun, 2021; Raj et al., 2016; Raj et al., 2021; Tanno et al., 2010; Zhu et al., 2024). The adjusted pooled estimate of LVEF was 2.98 (*P* < 0.01, I^2^ = 41%; Fig. 4B). Sensitivity analysis after adjustment showed that the result was stable (Fig. 4C). The funnel plot was symmetry, Begg’s test (*P* = 0.10) and Egger’s test (*P* = 0.07) revealed no publication bias (Fig. 4D). Moreover, seven studies (175 animals) were included in the meta-analysis of LVFS (Xuan et al., 2012; Lin et al., 2008; Liu et al., 2019; Mei, Liu & Sun, 2021; Tanno et al., 2010; Zhu et al., 2024). The pooled estimate of LVFS was 3.21 (*P* < 0.01, I^2^ = 71%; Fig. 4E) in the animal model of MI.

### Cardiac injury enzyme

After systematic data extraction from included studies, not enough data on cardiac injury enzymes were found in the MI model. Therefore, only the MIRI model was analyzed in this section. Serum biomarkers of myocardial injury, including LDH (13 studies, 210 animals) (Boshra, 2020; Cong et al., 2014; Dernek et al., 2004; Du et al., 2014; Feng et al., 2019; Hung et al., 2000; Kazemirad & Kazerani, 2020; Liao et al., 2015; Liu et al., 2023; Shen et al., 2012; Singh et al., 2024; Tian, Xiong & Xia, 2023; Hung, Su & Chen, 2004), CK-MB (11 studies, 180 animals) (Boshra, 2020; Dernek et al., 2004; Dong et al., 2015; Feng et al., 2019; Kazemirad & Kazerani, 2020; Liu et al., 2023; Manjunatha et al., 2020; Salian et al., 2024; Singh et al., 2024; Tian, Xiong & Xia, 2023; Xu et al., 2022), CK (three studies, 48 animals) (Cong et al., 2014; Liao et al., 2015; Hung, Su & Chen, 2004), and cTnT (three studies, 60 animals) (Boshra, 2020; Dernek et al., 2004; Dong et al., 2015) were analyzed.

The results revealed that RES treatment significantly reduced serum LDH (SMD: −9.52, *P* < 0.01, I^2^ = 85%), CK-MB (SMD: −10.96, *P* < 0.01, I^2^ = 90%), CK (SMD: −5.24, *P* = 0.01, I^2^ = 88%), and cTnT (SMD: −20.44, *P* = 0.02, I^2^ = 89%) in animal models of MIRI (Table 6 & Fig. S3). The sensitivity analysis in LDH and CK-MB revealed that the result was stable (Figs. S4A–S4B). The funnel plot of LDH and CK-MB was asymmetry with Begg’s test and Egger’s test, which suggested publication bias (Figs. S4C–S4D).

**Table 6:** The pooled estimate of cardiac injury enzyme and oxidative stress levels in MIRI and MI animal model.

**Moderator**	**N of studies**	**SMD**	**95% CI**	***P***	**I**^2^ (%)
**MIRI animal model**					
LDH	13	−9.52	−12.74, −6.31	<0.01	85
CK-MB	11	−10.96	−15.37, −6.65	<0.01	90
CK	3	−5.24	−9.26, −1.23	0.01	88
cTnT	3	−20.44	−38.19, −2.68	0.02	89
SOD	7	7.70	3.60, 11.79	<0.01	87
MDA	7	−4.17	−7.28, −1.06	<0.01	94
**MI animal model**					
SOD^*^	2	4.30	2.52, 6.08	<0.01	0
MDA	3	−6.89	−9.03, −4.76	<0.01	0

**Notes.**

LDH lactate dehydrogenase CK-MB creatine kinase isoenzyme CK creatine kinase cTnT cardiac troponin T SOD superoxide dismutase MDA malondialdehyde MIRI myocardial ischemia/reperfusion injury MI myocardial infarction

* Results for heart SOD in MI animal model were derived from only 2 studies with small sample sizes and should be considered exploratory.

### Oxidative stress levels

To determine the oxidative stress levels of the heart after RES treatment, SOD and MDA were selected as indicators for analysis.

In the MIRI models, six researches (108 animals) reported heart SOD (Boshra, 2020; Feng et al., 2019; Kazemirad & Kazerani, 2020; Salian et al., 2024; Shen et al., 2012; Xu et al., 2022), and seven researches (126 animals) reported heart MDA (Boshra, 2020; Dernek et al., 2004; Feng et al., 2019; Kazemirad & Kazerani, 2020; Ray et al., 1999; Shen et al., 2006; Shen et al., 2012). The results demonstrated that RES treatment significantly increased heart SOD (SMD: 7.70, *P* < 0.01, I^2^ = 87%), and reduced heart MDA (SMD: −4.17, *P* < 0.01, I^2^ = 94%) (Table 6 & Fig. S5).

In the MI models, two studies (22 animals) reported heart SOD (He et al., 2021; Xin et al., 2010), and three studies (34 animals) reported heart MDA (Liu et al., 2022; Ray et al., 1999; Xin et al., 2010). The results demonstrated that RES treatment significantly increased heart SOD (SMD: 4.30, *P* < 0.01, I^2^ = 0%), and reduced heart MDA (SMD: −6.89, *P* < 0.01, I^2^ = 0%) (Table 6 & Fig. S5). However, these findings are exploratory, as they were derived from a limited number of studies with small sample sizes, and should therefore be interpreted with caution.

### Inflammatory cytokine levels and apoptosis rate

**Figure 5:**
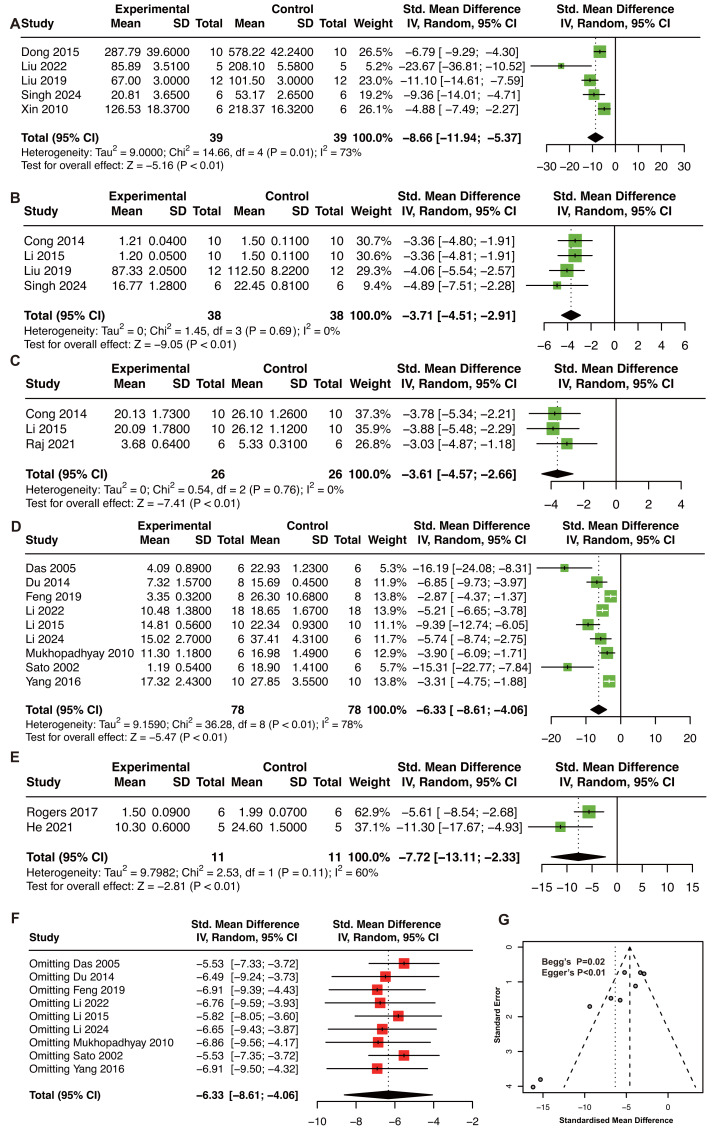
The pooled estimate of serum IL-1β. (A), serum TNF-α (B), and heart TNF-α (C) in MIRI and MI animal model. The pooled estimate of apoptosis rate in animal model of MIRI (D) and MI (E). Apoptosis results in MI models are based on only two studies with high heterogeneity and should be considered as exploratory. (F) Sensitivity analysis of apoptosis rate in animal model of MIRI. (G) Funnel plot, Begg’s test, and Egger’s test of apoptosis rate in animal model of MIRI. IL-1β, interleukin-1β; TNF-α, tumor necrosis factor-α; MIRI, myocardial ischemia/reperfusion injury; MI, myocardial infarction. (Dong et al., 2015; Liu et al., 2022; Liu et al., 2019; Singh et al., 2024; Xin et al., 2010; Cong et al., 2014; Li et al., 2015; Raj et al., 2021; Das et al., 2005; Du et al., 2014; Feng et al., 2019; Li, Duan & Shen, 2022; Li et al., 2024; Mukhopadhyay et al., 2010; Sato, Maulik & Das, 2002; Yang et al., 2016; Rogers & Otis, 2017; He et al., 2021).

Due to low heterogeneity within serum IL-1β, serum TNF-α, and heart TNF-α, the animal model of MIRI and MI were analyzed together. RES treatment significantly reduced serum IL-1β (five studies, SMD = −8.66, *P* < 0.01, I^2^ = 73%, Fig. 5A) (Dong et al., 2015; Liu et al., 2022; Liu et al., 2019; Singh et al., 2024; Xin et al., 2010), serum TNF-α (four studies, SMD = −3.71, *P* < 0.01, I^2^ = 0%, Fig. 5B) (Cong et al., 2014; Li et al., 2015; Liu et al., 2019; Singh et al., 2024), and cardiac TNF-α (three studies, SMD = −3.61, *P* < 0.01, I^2^ = 0%, Fig. 5C) (Cong et al., 2014; Li et al., 2015; Raj et al., 2021).

For apoptosis rate, nine studies (156 animals) in MIRI (Das et al., 2005; Du et al., 2014; Feng et al., 2019; Li, Duan & Shen, 2022; Li et al., 2015; Li et al., 2024; Mukhopadhyay et al., 2010; Sato, Maulik & Das, 2002; Yang et al., 2016), and two studies in MI (He et al., 2021; Rogers & Otis, 2017) models were included. RES treatment significantly decreased the apoptosis rate in both MIRI (SMD: −6.33, *P* < 0.01, I^2^ = 78%, Fig. 5D) and MI (SMD: −7.72, *P* < 0.01, I^2^ = 60%, Fig. 5E) models. Because of high heterogeneity (I^2^ = 60%) and the limited number of MI studies (*n* = 2), these results should be regarded as exploratory. The sensitivity analysis confirmed result stability (Fig. 5F). However, the funnel plot was asymmetry with Begg’s test (*P* = 0.048), and Egger’s test (*P* < 0.01), which indicated publication bias (Fig. 5G).

### Meta-regression and dosage-efficacy analysis

To further explore the sources of heterogeneity of IS in the MIRI model, univariate meta-regression analysis was performed (Table 7). Model type, IS measure methods, ischemia and reperfusion time, and duration did not significantly influence the effect sizes of IS. The drug delivery method accounted for 28.81% of between-study heterogeneity, with perfusion showing the largest reduction in IS (β = −2.64, *P* = 0.02). Animal species also contributed substantially to heterogeneity (*R*^2^ = 31.64%), with rabbits showing a worse effect size reduction.

**Table 7:** Meta-regression of infarct size in MIRI animal models.

**Moderator**	β	**se**	**z**	**Pval**	**R**^2^ (%)	**I**^2^ (%)	**QM Pval**
Model					0	84.82	0.34
I/R^*^							
ISO	1.84	1.94	0.95	0.34			
**IS measure methods**					2.7	82.49	0.40
IS/AAR^*^							
IS/LV	−0.40	1.13	0.35	0.72			
NR	−2.04	1.70	−1.20	0.22			
**Ischemia time**					0	85.25	0.53
≤30 min^*^							
30–60 min	−1.38	2.22	−0.62	0.53			
ISO	1.77	1.97	0.90	0.37			
**Reperfusion time**					7.49	81.46	0.17
≤1h^*^							
1–2 h	0.36	1.39	0.26	0.79			
>2 h	2.87	1.70	1.69	0.09			
ISO	2.59	2.17	1.19	0.23			
**Drug delivery**					28.81	78.03	0.01
Intraperitoneal^*^							
Oral	1.22	1.08	1.12	0.26			
Intravenous	−1.01	1.61	−0.62	0.53			
Perfusion	−2.64	1.20	−2.19	0.02			
**Duration**					0	84.33	0.34
30 min–1 day^*^						.	
≤30 min	−0.42	1.52	−0.28	0.78			
>1 day	1.17	1.50	0.79	0.43			
**Species**					31.64	76.80	0.005
Mice^*^							
Rats	−1.869	1.16	1.61	0.10			
Rabbits	4.51	2.34	1.93	0.05			

**Notes.**

* Reference, IS, infarction size; AAR, area at risk; LV, left ventricle; NR, not reported; ISO, isoprenaline; I/R, ischemia/reperfusion.

**Figure 6:**
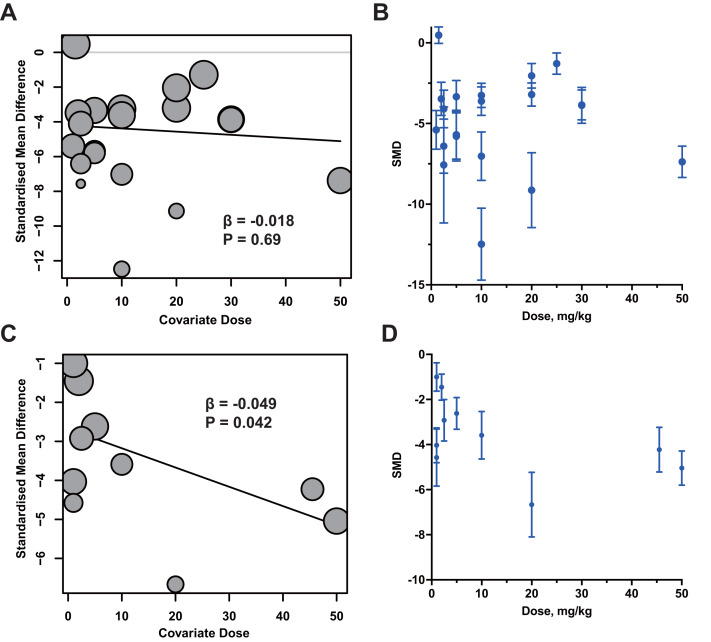
Meta-regression and dosage-efficacy analysis of oral administration RES in treatment of MIRI (A–B) and MI (C–D) animal models.

Due to the different administration methods, the dosage units of the drugs are inconsistent. Therefore, we conducted meta-regression and dosage-efficacy analysis on the oral and intraperitoneal RES dosage in MIRI and MI models. The results showed that the dose was not significantly associated with the overall effect size (β = −0.018, *P* = 0.69, Figs. 6A–6B) in MIRI models. Although, there is a certain correlation between the dose of RES and the therapeutic effect in the MI model (β = −0.049, *P* = 0.042, Figs. 6C–6D), a relatively low oral dose of RES appeared to produce comparable (10 mg/Kg in MIRI and 20 mg/Kg in MI models) therapeutic efficacy compared with higher doses in both MIRI and MI models. The meta-regression and dosage-efficacy analysis results further supporting no clear dose–response relationship between RES dosage and treatment efficacy of MIRI and MI.

## Discussion

### Summary of evidence

To investigate the role of RES in the fight against MI and MIRI, 57 articles with 1,125 animals were investigated in this meta-analysis. The results indicated that RES treatment lessened the IS, down-regulated serum biomarkers of myocardial injury (CK-MB and LDH), improved cardiac function parameters (LVEDP, dP/dt max, AF, and CF), increased heart SOD level, reduced heart MDA and serum inflammatory cytokines (IL-1β and TNF-α), and alleviated cardiomyocyte apoptosis in animal models of MI and MIRI. Moreover, sensitivity analysis indicated that the cardioprotective effect of RES was stable though with high heterogeneity. In summary, our results demonstrated that RES effectivity ameliorates MI and MIRI in pre-clinical animal studies.

### Molecular mechanisms

RES is a natural polyphenol compound, which exerts pharmacological activities such as anti-cardiovascular disease and anti-tumor. Previous studies have found that in the rat MIRI model, RES reduces myocardial cell necrosis by anti-oxidative stress and anti-free radicals and plays a protective role in MIRI by regulating the NO pathway and promoting NO release (Hung et al., 2002). In addition, RES also influences the occurrence and progression of tumors by regulating and controlling signal transduction pathways such as cell division and growth, apoptosis, inflammation, angiogenesis, and metastasis (Jang, Im & Kim, 2022).

### Anti-oxidative stress effects

Oxidative stress plays an important role in MIRI (Bugger & Pfeil, 2020). Previous studies indicated that in the rat model of MIRI, the mitochondrial function of myocardial cells is seriously damaged, and the production of ROS is abnormally increased, leading to the increase of MI area. RES is a kind of antioxidant found naturally in plants. It functions as an antioxidant primarily by decreasing the production of free radicals and lipid peroxidation, and activating the enzymes and other pathways involved in antioxidant defense (Muñoz Bernal et al., 2021). SIRT1, a versatile protein deacetylase, was reported to be down-regulated in the hypoxic-reoxygenation (H/R) injury of cardiomyocytes (Guo et al., 2016). RES protects cardiomyocytes from H_2_O_2_-induced oxidative stress by activating SIRT1 and mitochondrial biogenesis signaling pathways (Li et al., 2013). DJ-1 participates in the regulation of oxidative stress by maintaining the activity of mitochondrial complex I. RES protects H9c2 cardiomyocytes from H/R-induced oxidative stress damage through increased expression of DJ-1, mitochondrial translocation, and the active mitochondrial complex I (Li et al., 2013). RES has been shown to reduce LPS-induced myocardial cell damage by inhibiting nuclear factor erythroid 2-related factor 2 (Nrf2), which could induce endogenous antioxidant enzymes against oxidative stress (Hao et al., 2013). RES preconditioning also significantly improved mitochondrial function and enhanced the activities of antioxidant enzymes SOD and GSH-PX by activating NRF2/ARE signaling pathways to alleviate cardiomyocyte damage (Spanier et al., 2009; Cheng et al., 2015). Especially, myocardial Nrf2 signaling is impaired in diabetic rats, leading to further deterioration of MIRI, and RES preconditioning can relieve MIRI by increasing Nrf2 expression and reducing oxidative stress index in diabetic rats (Xu et al., 2019a). In addition, RES was shown to significantly reduce ROS generation and MPT pore opening in ATG mice hearts to protect cardiomyocytes from MIRI (Ananthakrishnan et al., 2009). In SD rats, chronic myocardial ischemia reduced the protein expression of KLF15, and daily treatment with RES stimulates the expression of the KLF15 gene, which improves cardiac function and enhances cardiac remodeling and facilitates the regulation of redox homeostasis (Rogers & Otis, 2017).

### Anti-inflammation effects

The inflammatory response is an important pathophysiological mechanism of MIRI injury (Schäfer et al., 2022). In animal models of MIRI, the levels of inflammatory cytokines were significantly increased, and early RES treatment effectively reduced MIRI by inhibiting the inflammatory response (Buja, 2023). RES preconditioning attenuates serum and myocardial TNF-α production, IS, and myocardial apoptosis in MIRI rats by inhibiting Toll-like receptor 4 (TLR4)/NF-κB signaling pathway (Li et al., 2015). In addition, the cardioprotective effect of RES on MIRI animal models also involves inhibition of the NLRP3 inflammasome pathway (Dong et al., 2015), and activation of the Nrf2/ARE pathway (Hao et al., 2013; Cheng et al., 2015).

### Anti-apoptosis effects

Apoptosis induced by MIRI is an important way to lead to myocardial cell death. RES exhibits an anti-apoptotic effect on H/R damage of H9c2 cells, and the specific mechanism is related to restoring SIRT1 activity in a DJ-1-dependent manner, thus reducing the level of p53 acetylation (Xu et al., 2019c). Voltage-dependent anion channel 1 (VDAC1), a protein located in the mitochondrial outer membrane, was downregulated in cardiomyocytes’ H/R injury (Liao et al., 2015). RES was proven to inhibit mitochondria-mediated myocardial apoptosis by deacetylating VDAC1 and increasing VDAC1 protein expression in the H/R cell model (Tong et al., 2017). In MIRI rats, RES treatment prior to the onset of reperfusion significantly improved cardiac systolic function and reduced IS. The specific mechanism is that RES transferred GSK-3beta from the cytoplasm to mitochondria *via* the cGMP/PKG pathway, and finally interacted with cyclophilin D to regulate MPT pore opening, thereby alleviating reperfusion myocardial injury (Xi et al., 2009b).

### Other mechanisms

Increased intracellular free Ca^2+^ plays a crucial role in MIRI. In H9c2 cells, RES preconditioning not only inhibited intracellular calcium aggregation by up-regulating sarcoplasmic reticulum stress protein Grp94 to reduce H/R-induced cell necrosis (Vitadello et al., 2003), but also alleviated H/R-induced calcium overload by inhibiting STIM1, thus inhibiting cardiomyocyte apoptosis and promoting cardiac function recovery after MIRI (Xu et al., 2019b). Moreover, RES can also induce autophagy by activating the mammalian target of the rapamycin complex 2 (mTORC2) pathway, thus protecting cardiomyocytes against H/R injury (Gurusamy et al., 2010).

### Implications

Many pharmacological agents have been shown to reduce MIRI in animal models, however, no cardioprotective agent has been routinely used for the treatment of clinical MIRI up to date (Zheng et al., 2017). Among various theories, the features of the pathophysiology of MIRI include vascular leakage, oxidative stress, the loss of energy substrates, leukocyte entrapment, inflammation, apoptosis, and mitochondrial dysfunction (Yang et al., 2019; Liu et al., 2017). RES exerts pharmacological properties including antioxidant, anti-inflammatory, and immuno-modulatory functions (Pannu & Bhatnagar, 2019). The findings of the present study suggested that RES can significantly reduce the IS in MIRI animal models, down-regulate serum CK-MB and LDH, improve cardiac function parameters, reduce inflammatory cytokines, and cardiomyocyte apoptosis rate.

Our meta-regression and dose-efficacy analysis suggest that RES can exert cardioprotective effects even at relatively low doses. The observation of efficacy at lower doses has important translational implications, as it may reduce the risk of potential off-target effects or toxicity associated with higher doses. However, the absorption and metabolism of RES is influenced by age, gut microbiota composition, diet, and co-administered medications, which are rarely considered in preclinical studies but are critical in clinical settings (Silva et al., 2023; McGonigle & Ruggeri, 2014). Therefore, it is important to note that the dose-efficacy relationship observed in animal studies may not directly extrapolate to humans due to differences in pharmacokinetics, bioavailability, and metabolism (Van der Worp et al., 2010). In addition, RES rapidly undergoes glucuronidation and sulfation in the intestine and liver, resulting in low systemic concentrations of the parent compound and significant differences in plasma levels of its metabolites (Yu, Jia & Ren, 2024; De Vries, Strydom & Steenkamp, 2021). The oral bioavailability of RES is low, and its widespread first pass metabolic elimination may severely limit its clinical application. To overcome these limitations, several strategies can be further explored: novel drug delivery systems (*e.g.*, nanoparticles, liposomes), prodrugs or derivatives with improved pharmacokinetics, and co-administration with bioenhancers such as piperine or quercetin to inhibit metabolic enzymes (De Vries, Strydom & Steenkamp, 2021).

In addition to its independent cardioprotective effects, RES may serve as a complementary therapy alongside established interventions such as β-blockers, ACE inhibitors, statins, and reperfusion strategies (Gal et al., 2021). Therefore, combining RES with standard treatments could potentially yield additive or synergistic cardioprotective effects, particularly in complex pathophysiological settings like MIRI or MI. Future studies should explore these combination strategies in clinically relevant models, including animals with comorbidities, to better inform translational potential and guide eventual clinical evaluation.

### Limitation

At first, the potential for publication bias cannot be ignored. Positive results within animal studies are more likely to be published, whereas negative findings may remain unpublished (Mlinarić, Horvat & Šupak Smolčić, 2017), leading to an overestimation of the cardioprotective effects of RES. This is supported by the observed asymmetry in funnel plots and statistically significant Egger’s and Begg’s tests, indicating that studies reporting substantial effects are overrepresented. While we attempted to address this by performing sensitivity, subgroup, and meta-regression analysis, residual bias may still influence the pooled estimates. Therefore, the efficacy of RES observed in this meta-analysis should be interpreted cautiously, and further well-designed studies, including those reporting null results, are needed to validate these effects.

Secondly, substantial heterogeneity across the majority of pooled analysis (I^2^ > 75%) was observed. High heterogeneity indicates that between-study differences account for most of the observed variability. In this case, the heterogeneity may come from multiple sources, including differences in animal species and strains, model type, RES dose, route and duration of administration, outcome definitions, sampling time points for enzyme and oxidative stress measurements, and methodological quality. Statistically, high I^2^ reduces the confidence one can place in a single pooled estimate and limits generalizability, therefore, we used random-effects models and report prediction intervals to convey the range of effects likely to be observed in new studies. We also performed subgroup analysis, leave-one-out sensitivity analysis, meta-regression, and dosage-efficacy analysis. While some moderators partially explained the heterogeneity, substantial residual heterogeneity remained. Therefore, although the overall direction of effect was consistent across analyses, the magnitude of pooled effects should be interpreted cautiously. Future preclinical studies should standardize key experimental parameters to reduce heterogeneity and strengthen translational inference.

Thirdly, it is important to note that many included studies exhibited methodological limitations, particularly in blinding and randomization. The absence of random allocation and blinded outcome assessment may lead to selection bias and observer bias, potentially exaggerating the apparent cardioprotective effects of RES. These biases could affect both the effect size estimates and the interpretation of heterogeneity. Future preclinical studies should rigorously implement randomization, allocation concealment, and blinding to improve the reliability and translational relevance of findings.

Finally, due to convenience and widely used of rodent models, the majority of included studies were conducted in young, healthy small rodents without other comorbidities. Large-animal models, such as pigs, can better replicate the cardiovascular anatomy, physiology, and pharmacokinetics of humans. Besides, MIRI or MI patients have more complex cardiovascular comorbidities, age-related physiological changes, and potential drug interactions, which may influence treatment responses in clinical practice. Therefore, although our meta-analysis suggested that RES has cardioprotective effects in animal models, these findings may not fully reflect the complex pathophysiology and treatment response of human patients. It is necessary to conduct preclinical studies using comorbidity- and large-animal models under ARRIVE (Percie du Sert et al., 2020), IMPACT (Lecour et al., 2021), and CAESAR (Fernández-Jiménez & Ibanez, 2015) guidelines in the future to validate the cardioprotective effects of RES and improve its clinical applicability.

## Conclusions

Our findings suggest that RES appears to confer cardioprotective effects in animal models of MIRI and MI, including reduction of IS and serum cardiac enzymes, and improvement of cardiac function. However, the translational potential of RES remains uncertain, and additional preclinical studies with standardized protocols, comorbid models, and eventual clinical trials are needed to confirm these results.

##  Supplemental Information

10.7717/peerj.20708/supp-1 Supplemental Information 1 Sensitivity analysis of infarction size in animal model of MIRI (A) and MI (B). Funnel plot, Begg’s test, and Egger’s test of infarction size in animal model of MIRI (C) and MI (D) MIRI, myocardial ischemia/reperfusion injury; MI, myocardial infarction.

10.7717/peerj.20708/supp-2 Supplemental Information 2 Sensitivity analysis of LVDP (A), dP/dt max (B), and CF (C) in animal model of MIRI. Funnel plot, Begg’s test, and Egger’s test of LVDP (D), dP/dt max (E), and CF (F) in animal model of MIRI LVDP, left ventricular developing pressure; dP/dT max, maximum 1st derivative of developed pressure; CF, coronary flow; MIRI, myocardial ischemia/reperfusion injury.

10.7717/peerj.20708/supp-3 Supplemental Information 3 The pooled estimate of LDH (A), CK-MB (B), CK (C), and cTnT (D) in MIRI animal model LDH, lactate dehydrogenase; CK-MB, creatine kinase isoenzyme; CK, creatine kinase; cTnT, cardiac troponin T; MIRI, myocardial ischemia/reperfusion injury.

10.7717/peerj.20708/supp-4 Supplemental Information 4 Sensitivity analysis of LDH (A) and CK-MB in animal model of MIRI. Funnel plot, Begg’s test, and Egger’s test of LDH (C) and CK-MB (D) in animal model of MIRI LDH, lactate dehydrogenase; CK-MB, creatine kinase isoenzyme; MIRI, myocardial ischemia/reperfusion injury.

10.7717/peerj.20708/supp-5 Supplemental Information 5 The pooled estimate of heart SOD (A) and MDA (B) in MIRI animal model. The pooled estimate of heart SOD (C) and MDA (D) in MI animal model SOD, superoxide dismutase; MDA, malondialdehyde; MIRI, myocardial ischemia/reperfusion injury; MI, myocardial infarction.

10.7717/peerj.20708/supp-6 Supplemental Information 6 PRISMA checklist

10.7717/peerj.20708/supp-7 Supplemental Information 7 Raw Data of this meta-analysis

10.7717/peerj.20708/supp-8 Supplemental Information 8 The intended audience for this study

## Additional Information and Declarations

### Data Availability

The following information was supplied regarding data availability: The data is available in the Supplementary File.

## References

[ref-1] Acipreste Hudson E, Campos de Paula HM, Coelho YL, Glanzmann N, Da Silva AD, Mendes da Silva LH, Dos Santos Pires AC (2022). The kinetics of formation of resveratrol-β-cyclodextrin-NH(2) and resveratrol analog-β-cyclodextrin-NH(2) supramolecular complexes. Food Chemistry.

[ref-2] Adam T, Sharp S, Opie LH, Lecour S (2013). Loss of cardioprotection with ischemic preconditioning in aging hearts: role of sirtuin 1?. Journal of Cardiovascular Pharmacology and Therapeutics.

[ref-3] Ahmet I, Tae HJ, Lakatta EG, Talan M (2016). Long-term low dose dietary resveratrol supplement reduces cardiovascular structural and functional deterioration in chronic heart failure in rats. Canadian Journal of Physiology and Pharmacology.

[ref-4] Ananthakrishnan R, Kaneko M, Hwang YC, Quadri N, Gomez T, Li Q, Caspersen C, Ramasamy R (2009). Aldose reductase mediates myocardial ischemia-reperfusion injury in part by opening mitochondrial permeability transition pore. American Journal of Physiology-Heart and Circulatory Physiology.

[ref-5] Boshra SA (2020). Resveratrol modulates miR-34a in cardiotoxicity induced by isoproterenol. Journal of Medicinal Food.

[ref-6] Bradamante S, Barenghi L, Piccinini F, Bertelli AA, De Jonge R, Beemster P, De Jong JW (2003). Resveratrol provides late-phase cardioprotection by means of a nitric oxide- and adenosine-mediated mechanism. European Journal of Pharmacology.

[ref-7] Bugger H, Pfeil K (2020). Mitochondrial ROS in myocardial ischemia reperfusion and remodeling. Biochimica Et Biophysica Acta Molecular Basis of Disease.

[ref-8] Buja LM (2023). Pathobiology of myocardial ischemia and reperfusion injury: models, modes, molecular mechanisms, modulation, and clinical applications. Cardiology in Review.

[ref-9] Burstein B, Maguy A, Clément R, Gosselin H, Poulin F, Ethier N, Tardif J-C, Hébert TE, Calderone A, Nattel S (2007). Effects of resveratrol (trans-3, 5 4’-trihydroxystilbene) treatment on cardiac remodeling following myocardial infarction. Journal of Pharmacology and Experimental Therapeutics.

[ref-10] Cai X, Yang C, Shao L, Zhu H, Wang Y, Huang X, Wang S, Hong L (2020). Targeting NOX 4 by petunidin improves anoxia/reoxygenation-induced myocardium injury. European Journal of Pharmacology.

[ref-11] Chang JC, Lien CF, Lee WS, Chang HR, Hsu YC, Luo YP, Jeng JR, Hsieh JC, Yang KT (2019). Intermittent hypoxia prevents myocardial mitochondrial Ca(2+) overload and cell death during ischemia/reperfusion: the role of reactive oxygen species. Cell.

[ref-12] Cheng L, Jin Z, Zhao R, Ren K, Deng C, Yu S (2015). Resveratrol attenuates inflammation and oxidative stress induced by myocardial ischemia-reperfusion injury: role of Nrf2/ARE pathway. International Journal of Clinical and Experimental Medicine.

[ref-13] Cong X, Li Y, Lu N, Dai Y, Zhang H, Zhao X, Liu Y (2014). Resveratrol attenuates the inflammatory reaction induced by ischemia/reperfusion in the rat heart. Molecular Medicine Reports.

[ref-14] Das S, Alagappan VKT, Bagchi D, Sharma HS, Maulik N, Das DK (2005). Coordinated induction of iNOS-VEGF-KDR-eNOS after resveratrol consumption—a potential mechanism for resveratrol preconditioning of the heart. Vascular Pharmacology.

[ref-15] Das S, Falchi M, Bertelli A, Maulik N, Das DK (2006). Attenuation of ischemia/reperfusion injury in rats by the anti-inflammatory action of resveratrol. Arzeneimittel-Forschung.

[ref-16] Dernek S, Ikizler M, Erkasap N, Ergun B, Koken T, Yilmaz K, Sevin B, Kaygisiz Z, Kural T (2004). Cardioprotection with resveratrol pretreatment: improved beneficial effects over standard treatment in rat hearts after global ischemia. Scandinavian Cardiovascular Journal.

[ref-17] De Vries K, Strydom M, Steenkamp V (2021). A brief updated review of advances to enhance resveratrol’s bioavailability. Molecules.

[ref-18] Dong W, Yang R, Yang J, Yang J, Ding J, Wu H, Zhang J (2015). Resveratrol pretreatment protects rat hearts from ischemia/reperfusion injury partly *via* a NALP3 inflammasome pathway. International Journal of Clinical and Experimental Pathology.

[ref-19] Du R-H, Dai T, Cao W-J, Lu M, Ding J-H, Hu G (2014). Kir6.2-containing ATP-sensitive K(+) channel is required for cardioprotection of resveratrol in mice. Cardiovascular Diabetology.

[ref-20] Feng L, Ren J, Li Y, Yang G, Kang L, Zhang S, Ma C, Li J, Liu J, Yang L, Qi Z (2019). Resveratrol protects against isoproterenol induced myocardial infarction in rats through VEGF-B/AMPK/eNOS/NO signalling pathway. Free Radical Research.

[ref-21] Fernández-Jiménez R, Ibanez B (2015). CAESAR: one step beyond in the construction of a translational bridge for cardioprotection. Circulation Research.

[ref-22] Frantz S, Hundertmark MJ, Schulz-Menger J, Bengel FM, Bauersachs J (2022). Left ventricular remodelling post-myocardial infarction: pathophysiology, imaging, and novel therapies. European Heart Journal.

[ref-23] Gal R, Deres L, Toth K, Halmosi R, Habon T (2021). The effect of resveratrol on the cardiovascular system from molecular mechanisms to clinical results. International Journal of Molecular Sciences.

[ref-24] Gál R, Halmosi R, Gallyas Jr F, Tschida M, Mutirangura P, Tóth K, Alexy T, Czopf L (2023). Resveratrol and beyond: the effect of natural polyphenols on the cardiovascular system: a narrative review. Biomedicines.

[ref-25] Goh SS, Woodman OL, Pepe S, Cao AH, Qin C, Ritchie RH (2007). The red wine antioxidant resveratrol prevents cardiomyocyte injury following ischemia-reperfusion *via* multiple sites and mechanisms. Antioxidants & Redox Signaling.

[ref-26] Gu XS, Wang ZB, Ye Z, Lei JP, Li L, Su DF, Zheng X (2014). Resveratrol, an activator of SIRT1, upregulates AMPK and improves cardiac function in heart failure. Genetics and Molecular Research.

[ref-27] Guo Y, Zhang L, Li F, Hu CP, Zhang Z (2016). Restoration of sirt1 function by pterostilbene attenuates hypoxia-reoxygenation injury in cardiomyocytes. European Journal of Pharmacology.

[ref-28] Gurusamy N, Lekli I, Mukherjee S, Ray D, Ahsan MK, Gherghiceanu M, Popescu LM, Das DK (2010). Cardioprotection by resveratrol: a novel mechanism *via* autophagy involving the mTORC2 pathway. Cardiovascular Research.

[ref-29] Hale SL, Kloner RA (2001). Effects of resveratrol, a flavinoid found in red wine, on infarct size in an experimental model of ischemia/reperfusion. Journal of Studies on Alcohol.

[ref-30] Hao E, Lang F, Chen Y, Zhang H, Cong X, Shen X, Su G (2013). Resveratrol alleviates endotoxin-induced myocardial toxicity *via* the Nrf2 transcription factor. PLOS ONE.

[ref-31] He Y, Lu X, Chen T, Yang Y, Zheng J, Chen C, Zhang Y, Lei W (2021). Resveratrol protects against myocardial ischemic injury *via* the inhibition of NF-κB-dependent inflammation and the enhancement of antioxidant defenses. International Journal of Molecular Medicine.

[ref-32] Hooijmans CR, Rovers MM, De Vries RBM, Leenaars M, Ritskes-Hoitinga M, Langendam MW (2014). SYRCLE’s risk of bias tool for animal studies. BMC Medical Research Methodology.

[ref-33] Hung LM, Chen JK, Huang SS, Lee RS, Su MJ (2000). Cardioprotective effect of resveratrol, a natural antioxidant derived from grapes. Cardiovascular Research.

[ref-34] Hung LM, Su MJ, Chen JK (2004). Resveratrol protects myocardial ischemia-reperfusion injury through both NO-dependent and NO-independent mechanisms. Free Radical Biology and Medicine.

[ref-35] Hung LM, Su MJ, Chu WK, Chiao CW, Chan WF, Chen JK (2002). The protective effect of resveratrols on ischaemia-reperfusion injuries of rat hearts is correlated with antioxidant efficacy. British Journal of Pharmacology.

[ref-36] Jang JY, Im E, Kim ND (2022). Mechanism of resveratrol-induced programmed cell death and new drug discovery against cancer: a review. International Journal of Molecular Sciences.

[ref-37] Jensen RV, Hjortbak MV, Bøtker HE (2020). Ischemic heart disease: an update. Seminars in Nuclear Medicine.

[ref-38] Jin X, Wang X, Sun J, Tan W, Zhang G, Han J, Xie M, Zhou L, Yu Z, Xu T, Wang C, Wang Y, Zhou X, Jiang H (2022). Subthreshold splenic nerve stimulation prevents myocardial ischemia-reperfusion injury *via* neuroimmunomodulation of proinflammatory factor levels. International Immunopharmacology.

[ref-39] Kaga S, Zhan LJ, Matsumoto M, Maulik N (2005). Resveratrol enhances neovascularization in the infarcted rat myocardium through the induction of thioredoxin-1, heme oxygenase-1 and vascular endothelial growth factor. Journal of Molecular and Cellular Cardiology.

[ref-40] Kanamori H, Takemura G, Goto K, Tsujimoto A, Ogino A, Takeyama T, Kawaguchi T, Watanabe T, Morishita K, Kawasaki M, Mikami A, Fujiwara T, Fujiwara H, Seishima M, Minatoguchi S (2013). Resveratrol reverses remodeling in hearts with large, old myocardial infarctions through enhanced autophagy-activating AMP kinase pathway. American Journal of Pathology.

[ref-41] Kazemirad H, Kazerani HR (2020). Cardioprotective effects of resveratrol following myocardial ischemia and reperfusion. Molecular Biology Reports.

[ref-42] Lamont KT, Somers S, Lacerda L, Opie LH, Lecour S (2011). Is red wine a SAFE sip away from cardioprotection? Mechanisms involved in resveratrol- and melatonin-induced cardioprotection. Journal of Pineal Research.

[ref-43] Lecour S, Andreadou I, Bøtker HE, Davidson SM, Heusch G, Ruiz-Meana M, Schulz R, Zuurbier CJ, Ferdinandy P, Hausenloy DJ (2021). IMproving preclinical assessment of cardioprotective therapies (IMPACT) criteria: guidelines of the EU-CARDIOPROTECTION COST action. Basic Research in Cardiology.

[ref-44] Lekli I, Ray D, Mukherjee S, Gurusamy N, Ahsan MK, Juhasz B, Bak I, Tosaki A, Gherghiceanu M, Popescu LM, Das DK (2010). Co-ordinated autophagy with resveratrol and gamma-tocotrienol confers synergetic cardioprotection. Journal of Cellular and Molecular Medicine.

[ref-45] Lekli I, Szabo G, Juhasz B, Das S, Das M, Varga E, Szendrei L, Gesztelyi R, Varadi J, Bak I, Das DK, Tosaki A (2008). Protective mechanisms of resveratrol against ischemia-reperfusion-induced damage in hearts obtained from Zucker obese rats: the role of GLUT-4 and endothelin. American Journal of Physiology-Heart and Circulatory Physiology.

[ref-46] Li J, Duan Q-J, Shen J (2022). Resveratrol pretreatment improves mitochondrial function and alleviates myocardial ischemia-reperfusion injury by up-regulating miR-20b-5p to inhibit STIM2. Zhongguo Zhong Yao Za Zhi.

[ref-47] Li L, Wang J, Zhang D, Deng L, Zhao X, Wang C, Yan X, Hu S (2024). Resveratrol relieves myocardial ischemia-reperfusion injury through inhibiting AKT nitration modification. Redox Report.

[ref-48] Li J, Xie C, Zhuang J, Li H, Yao Y, Shao C, Wang H (2015). Resveratrol attenuates inflammation in the rat heart subjected to ischemia-reperfusion: role of the TLR4/NF-κB signaling pathway. Molecular Medicine Reports.

[ref-49] Li YG, Zhu W, Tao JP, Xin P, Liu MY, Li JB, Wei M (2013). Resveratrol protects cardiomyocytes from oxidative stress through SIRT1 and mitochondrial biogenesis signaling pathways. Biochemical and Biophysical Research Communications.

[ref-50] Liao Z, Liu D, Tang L, Yin D, Yin S, Lai S, Yao J, He M (2015). Long-term oral resveratrol intake provides nutritional preconditioning against myocardial ischemia/reperfusion injury: involvement of VDAC1 downregulation. Molecular Nutrition & Food Research.

[ref-51] Lin JF, Lin SM, Chih CL, Nien MW, Su HH, Hu BR, Huang SS, Tsai SK (2008). Resveratrol reduces infarct size and improves ventricular function after myocardial ischemia in rats. Life Sciences.

[ref-52] Liu S, Du Y, Shi K, Yang Y, Yang Z (2019). Resveratrol improves cardiac function by promoting M2-like polarization of macrophages in mice with myocardial infarction. American Journal of Translational Research.

[ref-53] Liu S, Ren J, Liu S, Zhao X, Liu H, Zhou T, Wang X, Liu H, Tang L, Chen H (2023). Resveratrol inhibits autophagy against myocardial ischemia-reperfusion injury through the DJ-1/MEKK1/JNK pathway. European Journal of Pharmacology.

[ref-54] Liu H, Wang C, Qiao Z, Xu Y (2017). Protective effect of curcumin against myocardium injury in ischemia reperfusion rats. Pharmaceutical Biology.

[ref-55] Liu J, Zhang M, Qin C, Wang Z, Chen J, Wang R, Hu J, Zou Q, Niu X (2022). Resveratrol attenuate myocardial injury by inhibiting ferroptosis *via* inducing KAT5/GPX4 in myocardial infarction. Frontiers in Pharmacology.

[ref-56] Manjunatha S, Shaik AH, Prasad ME, Al Omar SY, Mohammad A, Kodidhela LD (2020). Combined cardio-protective ability of syringic acid and resveratrol against isoproterenol induced cardio-toxicity in rats *via* attenuating NF-kB and TNF-alpha pathways. Scientific Reports.

[ref-57] Mao ZJ, Lin H, Hou JW, Zhou Q, Wang Q, Chen YH (2019). A meta-analysis of resveratrol protects against myocardial ischemia/reperfusion injury: evidence from small animal studies and insight into molecular mechanisms. Oxidative Medicine and Cellular Longevity.

[ref-58] Matsumura N, Takahara S, Maayah ZH, Parajuli N, Byrne NJ, Shoieb SM, Soltys C-LM, Beker DL, Masson G, El-Kadi AOS, Dyck JRB (2018). Resveratrol improves cardiac function and exercise performance in MI-induced heart failure through the inhibition of cardiotoxic HETE metabolites. Journal of Molecular and Cellular Cardiology.

[ref-59] McGonigle P, Ruggeri B (2014). Animal models of human disease: challenges in enabling translation. Biochemical Pharmacology.

[ref-60] Mei C, Liu W, Sun P (2021). Resveratrol inhibits myocardial apoptosis by regulating the protein kinase R-like endoplasmic reticulum kinase endoplasmic reticulum stress pathway and improves myocardial remodeling and cardiac function after myocardial infarction. Indian Journal of Pharmaceutical Sciences.

[ref-61] Mlinarić A, Horvat M, Šupak Smolčić V (2017). Dealing with the positive publication bias: why you should really publish your negative results. Biochemical Medicine.

[ref-62] Mukhopadhyay P, Mukherjee S, Ahsan K, Bagchi A, Pacher P, Das DK (2010). Restoration of altered microRNA expression in the ischemic heart with resveratrol. PLOS ONE.

[ref-63] Muñoz Bernal ÓA, Coria-Oliveros AJ, De la Rosa LA, Rodrigo-García J, Del Rocío Martínez-Ruiz N, Sayago-Ayerdi SG, Alvarez-Parrilla E (2021). Cardioprotective effect of red wine and grape pomace. Food Research International.

[ref-64] Naumenko SE, Latysheva TV, Gilinsky MA, Rogachev AD, Komarova NI, Salakhutdinov NF, Tolstikov GA (2013). Cardioprotective effect of resveratrol and resveratroloside. Cardiovascular and Hematological Agents in Medicinal Chemistry.

[ref-65] Ou W, Liang Y, Qin Y, Wu W, Xie M, Zhang Y, Zhang Y, Ji L, Yu H, Li T (2021). Hypoxic acclimation improves cardiac redox homeostasis and protects heart against ischemia-reperfusion injury through upregulation of O-GlcNAcylation. Redox Biology.

[ref-66] Pannu N, Bhatnagar A (2019). Resveratrol: from enhanced biosynthesis and bioavailability to multitargeting chronic diseases. Biomedicine and Pharmacotherapy.

[ref-67] Penumathsa SV, Thirunavukkarasu M, Koneru S, Juhasz B, Zhan L, Pant R, Menon VP, Otani H, Maulik N (2007). Statin and resveratrol in combination induces cardioprotection against myocardial infarction in hypercholesterolemic rat. Journal of Molecular and Cellular Cardiology.

[ref-68] Percie du Sert N, Hurst V, Ahluwalia A, Alam S, Avey MT, Baker M, Browne WJ, Clark A, Cuthill IC, Dirnagl U, Emerson M, Garner P, Holgate ST, Howells DW, Karp NA, Lazic SE, Lidster K, MacCallum CJ, Macleod M, Pearl EJ, Petersen OH, Rawle F, Reynolds P, Rooney K, Sena ES, Silberberg SD, Steckler T, Würbel H (2020). The ARRIVE guidelines 2.0: updated guidelines for reporting animal research. PLOS Biology.

[ref-69] Raj P, Aloud BM, Louis XL, Yu L, Zieroth S, Netticadan T (2016). Resveratrol is equipotent to perindopril in attenuating post-infarct cardiac remodeling and contractile dysfunction in rats. Journal of Nutritional Biochemistry.

[ref-70] Raj P, Louis XL, Thandapilly SJ, Movahed A, Zieroth S, Netticadan T (2014). Potential of resveratrol in the treatment of heart failure. Life Sciences.

[ref-71] Raj P, Sayfee K, Parikh M, Yu L, Wigle J, Netticadan T, Zieroth S (2021). Comparative and combinatorial effects of resveratrol and sacubitril/valsartan alongside valsartan on cardiac remodeling and dysfunction in MI-induced rats. Molecules.

[ref-72] Rao SV, O’Donoghue ML, Ruel M, Rab T, Tamis-Holland JE, Alexander JH, Baber U, Baker H, Cohen MG, Cruz-Ruiz M, Davis LL, De Lemos JA, DeWald TA, Elgendy IY, Feldman DN, Goyal A, Isiadinso I, Menon V, Morrow DA, Mukherjee D, Platz E, Promes SB, Sandner S, Sandoval Y, Schunder R, Shah B, Stopyra JP, Talbot AW, Taub PR, Williams MS (2025). ACC/AHA/ACEP/NAEMSP/SCAI guideline for the management of patients with acute coronary syndromes: a report of the American College of Cardiology/American Heart Association Joint Committee on clinical practice guidelines. Circulation.

[ref-73] Ray PS, Maulik G, Cordis GA, Bertelli AA, Bertelli A, Das DK (1999). The red wine antioxidant resveratrol protects isolated rat hearts from ischemia reperfusion injury. Free Radical Biology and Medicine.

[ref-74] Rodrigo R, Retamal C, Schupper D, Vergara-Hernández D, Saha S, Profumo E, Buttari B, Saso L (2022). Antioxidant cardioprotection against reperfusion injury: potential therapeutic roles of resveratrol and quercetin. Molecules.

[ref-75] Rogers RG, Otis JS (2017). Resveratrol-mediated expression of KLF15 in the ischemic myocardium is associated with an improved cardiac phenotype. Cardiocasular Drugs and Therapy.

[ref-76] Roth GA, Mensah GA, Johnson CO, Addolorato G, Ammirati E, Baddour LM, Barengo NC, Beaton AZ, Benjamin EJ, Benziger CP, Bonny A, Brauer M, Brodmann M, Cahill TJ, Carapetis J, Catapano AL, Chugh SS, Cooper LT, Coresh J, Criqui M, DeCleene N, Eagle KA, Emmons-Bell S, Feigin VL, Fernández-Solá J, Fowkes G, Gakidou E, Grundy SM, He FJ, Howard G, Hu F, Inker L, Karthikeyan G, Kassebaum N, Koroshetz W, Lavie C, Lloyd-Jones D, Lu HS, Mirijello A, Temesgen AM, Mokdad A, Moran AE, Muntner P, Narula J, Neal B, Ntsekhe M, Moraes de Oliveira G, Otto C, Owolabi M, Pratt M, Rajagopalan S, Reitsma M, Ribeiro ALP, Rigotti N, Rodgers A, Sable C, Shakil S, Sliwa-Hahnle K, Stark B, Sundström J, Timpel P, Tleyjeh IM, Valgimigli M, Vos T, Whelton PK, Yacoub M, Zuhlke L, Murray C, Fuster V (2020). Global burden of cardiovascular diseases and risk factors, 1990–2019: update from the GBD 2019 study. Journal of the American College of Cardiology.

[ref-77] Salian TR, Noushida N, Gowda BHJ, Chakraborty M, Srinivasulu SPE, Ahmed MG (2024). Cardioprotective potential of resveratrol alone and in combination with piperine on isoproterenol-induced myocardial infarction in rat: investigation on oral bioavailability of resveratrol. International Journal of Pharmaceutical Investigation.

[ref-78] Sato M, Maulik N, Das DK (2002). Cardioprotection with alcohol: role of both alcohol and polyphenolic antioxidants. Annals of the New York Academy of Sciences.

[ref-79] Schäfer A, König T, Bauersachs J, Akin M (2022). Novel therapeutic strategies to reduce reperfusion injury after acute myocardial infarction. Current Problems in Cardiology.

[ref-80] Shalwala M, Zhu S-G, Das A, Salloum FN, Xi L, Kukreja RC (2014). Sirtuin 1 (SIRT1) activation mediates sildenafil induced delayed cardioprotection against ischemia-reperfusion injury in mice. PLOS ONE.

[ref-81] Shen M, Jia G-L, Wang Y-M, Ma H (2006). Cardioprotective effect of resvaratrol pretreatment on myocardial ischemia-reperfusion induced injury in rats. Vascular Pharmacology.

[ref-82] Shen M, Wu R-X, Zhao L, Li J, Guo H-T, Fan R, Cui Y, Wang Y-M, Yue S-Q, Pei J-M (2012). Resveratrol attenuates ischemia/reperfusion injury in neonatal cardiomyocytes and its underlying mechanism. PLOS ONE.

[ref-83] Shi A, Zeng Y, Xin D, Zhou Y, Zhao L, Peng J (2022). Real-time visualization of the antioxidative potency of drugs for the prevention of myocardium ischemia-reperfusion injury by a NIR fluorescent nanoprobe. ACS Sensors.

[ref-84] Silva PM, Gonçalves C, Pastrana LM, Coimbra MA, Vicente AA, Cerqueira MA (2023). Recent advances in oral delivery systems of resveratrol: foreseeing their use in functional foods. Food & Function.

[ref-85] Singh J, Bisht P, Srivastav S, Kumar Y, Sharma V, Kumar A, Akhtar MS, Khan MF, Aldosari SA, Yadav S, Yadav NK, Mukherjee M, Sharma AK (2024). Amelioration of endothelial integrity by 3, 5, 4′-trihydroxy-trans-stilbene against high-fat-diet-induced obesity and -associated vasculopathy and myocardial infarction in rats, targeting TLR4/MyD88/NF-κB/iNOS signaling cascade. Biochemical and Biophysical Research Communications.

[ref-86] Soltan F, Esmaili Dahej M, Yadegari M, Moradi A, Hafizin Barjin Z, Safari F (2021). Resveratrol confers protection against ischemia/reperfusion injury by increase of angiotensin (1-7) expression in a rat model of myocardial hypertrophy. Journal of Cardiovascular Pharmacology.

[ref-87] Spanier G, Xu H, Xia N, Tobias S, Deng S, Wojnowski L, Forstermann U, Li H (2009). Resveratrol reduces endothelial oxidative stress by modulating the gene expression of superoxide dismutase 1 (SOD1), glutathione peroxidase 1 (GPx1) and NADPH oxidase subunit (Nox4). Journal of Physiology and Pharmacology.

[ref-88] Tanno M, Kuno A, Yano T, Miura T, Hisahara S, Ishikawa S, Shimamoto K, Horio Y (2010). Induction of manganese superoxide dismutase by nuclear translocation and activation of SIRT1 promotes cell survival in chronic heart failure. Journal of Biological Chemistry.

[ref-89] Teimouri M, Homayouni-Tabrizi M, Rajabian A, Amiri H, Hosseini H (2022). Anti-inflammatory effects of resveratrol in patients with cardiovascular disease: a systematic review and meta-analysis of randomized controlled trials. Complementary Therapies in Medicine.

[ref-90] Thuc LC, Teshima Y, Takahashi N, Nishio S, Fukui A, Kume O, Saito S, Nakagawa M, Saikawa T (2012). Inhibition of Na^+^ -H^+^ exchange as a mechanism of rapid cardioprotection by resveratrol. British Journal of Pharmacology.

[ref-91] Tian H, Xiong Y, Xia Z (2023). Resveratrol ameliorates myocardial ischemia/reperfusion induced necroptosis through inhibition of the Hippo pathway. Journal of Bioenergetics and Biomembranes.

[ref-92] Tong Z, Xie Y, He M, Ma W, Zhou Y, Lai S, Meng Y, Liao Z (2017). VDAC1 deacetylation is involved in the protective effects of resveratrol against mitochondria-mediated apoptosis in cardiomyocytes subjected to anoxia/reoxygenation injury. Biomedicine & Pharmacotherapy = Biomedecine & Pharmacotherapie.

[ref-93] Van der Worp HB, Howells DW, Sena ES, Porritt MJ, Rewell S, O’Collins V, Macleod MR (2010). Can animal models of disease reliably inform human studies?. PLOS Medicine.

[ref-94] Vitadello M, Penzo D, Petronilli V, Michieli G, Gomirato S, Menabò R, Di Lisa F, Gorza L (2003). Overexpression of the stress protein Grp94 reduces cardiomyocyte necrosis due to calcium overload and simulated ischemia. The FASEB Journal.

[ref-95] Wang J, Liu Y, Liu Y, Huang H, Roy S, Song Z, Guo B (2022). Recent advances in nanomedicines for imaging and therapy of myocardial ischemia-reperfusion injury. Journal of Controlled Release.

[ref-96] Xi J, Wang H, Mueller RA, Norfleet EA, Xu Z (2009a). Mechanism for resveratrol-induced cardioprotection against reperfusion injury involves glycogen synthase kinase 3beta and mitochondrial permeability transition pore. European Journal of Pharmacology.

[ref-97] Xi J, Wang H, Mueller RA, Norfleet EA, Xu Z (2009b). Mechanism for resveratrol-induced cardioprotection against reperfusion injury involves glycogen synthase kinase 3beta and mitochondrial permeability transition pore. European Journal of Pharmacology.

[ref-98] Xie S, Xing Y, Shi W, Zhang M, Chen M, Fang W, Liu S, Zhang T, Zeng X, Chen S, Wang S, Deng W, Tang Q (2022). Cardiac fibroblast heat shock protein 47 aggravates cardiac fibrosis post myocardial ischemia-reperfusion injury by encouraging ubiquitin specific peptidase 10 dependent Smad4 deubiquitination. Acta Pharmaceutica Sinica B.

[ref-99] Xin P, Pan Y, Zhu W, Huang S, Wei M, Chen C (2010). Favorable effects of resveratrol on sympathetic neural remodeling in rats following myocardial infarction. European Journal of Pharmacology.

[ref-100] Xing Z, He Q, Xiong Y, Zeng X (2021). Systematic pharmacology reveals the antioxidative stress and anti-inflammatory mechanisms of resveratrol intervention in myocardial ischemia-reperfusion injury. Evidence-Based Complementary and Alternative Medicine.

[ref-101] Xu H, Cheng J, Wang X, Liu H, Wang S, Wu J, Xu B, Chen A, He F (2019b). Resveratrol pretreatment alleviates myocardial ischemia/reperfusion injury by inhibiting STIM1-mediated intracellular calcium accumulation. Journal of Physiology and Biochemistry.

[ref-102] Xu G, Ma Y, Jin J, Wang X (2022). Activation of AMPK/p38/Nrf2 is involved in resveratrol alleviating myocardial ischemia-reperfusion injury in diabetic rats as an endogenous antioxidant stress feedback. Annals of Translational Medicine.

[ref-103] Xu RY, Xu XW, Deng YZ, Ma ZX, Li XR, Zhao L, Qiu LJ, Liu HY, Chen HP (2019c). Resveratrol attenuates myocardial hypoxia/reoxygenation-induced cell apoptosis through DJ-1-mediated SIRT1-p53 pathway. Biochemical and Biophysical Research Communications.

[ref-104] Xu G, Zhao X, Fu J, Wang X (2019a). Resveratrol increase myocardial Nrf2 expression in type 2 diabetic rats and alleviate myocardial ischemia/reperfusion injury (MIRI). Annals of Palliative Medicine.

[ref-105] Xuan W, Wu B, Chen C, Chen B, Zhang W, Xu D, Bin J, Liao Y (2012). Resveratrol improves myocardial ischemia and ischemic heart failure in mice by antagonizing the detrimental effects of fractalkine. Critical Care Medicine.

[ref-106] Yang M, Linn BS, Zhang Y, Ren J (2019). Mitophagy and mitochondrial integrity in cardiac ischemia-reperfusion injury. Biochimica et Biophysica Acta—Molecular Basis of Disease.

[ref-107] Yang L, Zhang Y, Zhu M, Zhang Q, Wang X, Wang Y, Zhang J, Li J, Yang L, Liu J, Liu F, Yang Y, Kang L, Shen Y, Qi Z (2016). Resveratrol attenuates myocardial ischemia/reperfusion injury through up-regulation of vascular endothelial growth factor B. Free Radical Biology and Medicine.

[ref-108] Yu X, Jia Y, Ren F (2024). Multidimensional biological activities of resveratrol and its prospects and challenges in the health field. Frontiers in Nutrition.

[ref-109] Zeng YF, Guo QH, Wei XY, Chen SY, Deng S, Liu JJ, Yin N, Liu Y, Zeng WJ (2023). Cardioprotective effect of curcumin on myocardial ischemia/reperfusion injury: a meta-analysis of preclinical animal studies. Frontiers in Pharmacology.

[ref-110] Zheng Q, Bao XY, Zhu PC, Tong Q, Zheng GQ, Wang Y (2017). Ginsenoside Rb1 for myocardial ischemia/reperfusion injury: preclinical evidence and possible mechanisms. Oxidative Medicine and Cellular Longevity.

[ref-111] Zhu X, Ma E, Ge Y, Yuan M, Guo X, Peng J, Zhu W, Ren D-N, Wo D (2024). Resveratrol protects against myocardial ischemic injury in obese mice *via* activating SIRT3/FOXO3a signaling pathway and restoring redox homeostasis. Biomedicine & Pharmacotherapy.

