# Supplementary material for: Cardioprotective effect of resveratrol in myocardial ischemia/reperfusion injury and myocardial infarction: a pre-clinical meta-analysis in animal studies
Source: PeerJ. 2026 Feb 4;14:e20708. doi: 10.7717/peerj.20708 (PMC12882730; doi:10.7717/peerj.20708)
Supplement: Supplemental Information 8 [file peerj-14-20708-s008.docx]

**The intended audience for this study includes:**

• Cardiovascular researchers and clinicians: Particularly those interested in cardioprotective strategies, myocardial ischemia/reperfusion (I/R) injury, myocardial infarction (MI), and pre-clinical drug development.

• Pharmacologists and experimental scientists: Professionals focused on natural compounds, polyphenols, and resveratrol as potential therapeutic agents for cardiovascular diseases.

• Translational medicine specialists: Researchers aiming to bridge findings from animal studies to clinical applications, especially in the field of cardioprotection.

• Graduate students and academicians: Those engaged in cardiovascular pathophysiology, meta-analysis methodology, and pre-clinical study design.

• Healthcare policymakers and industry stakeholders: Interested in emerging evidence on natural compounds for cardiovascular disease prevention and therapy.
